# Metformin Reduces the Senescence of Renal Tubular Epithelial Cells in Diabetic Nephropathy via the MBNL1/miR-130a-3p/STAT3 Pathway

**DOI:** 10.1155/2020/8708236

**Published:** 2020-02-10

**Authors:** Xue Jiang, Xue-lei Ruan, Yi-xue Xue, Shuang Yang, Mai Shi, Li-ning Wang

**Affiliations:** ^1^Department of Nephrology, The First Hospital of China Medical University, Shenyang 110001, China; ^2^Department of Neurobiology, School of Life Sciences, China Medical University, Shenyang 110122, China; ^3^Key Laboratory of Cell Biology, Ministry of Public Health of China, China Medical University, Shenyang 110122, China; ^4^Key Laboratory of Medical Cell Biology, Ministry of Education of China, China Medical University, Shenyang 110122, China

## Abstract

Senescence of renal tubular epithelial cells plays an important role in diabetic nephropathy, but the mechanism is unknown. Metformin may alleviate diabetic nephropathy by reducing this senescence. This study is aimed at clarifying the effects and mechanism of metformin on the senescence of renal tubular epithelial cells in diabetic nephropathy. We found that metformin reduced the expression of senescence-associated gene P21 in high-glucose-induced (30 mmol/L) renal tubular epithelial cells and decreased the *β*-galactosidase positive staining rate (decreased 16%, *p* < 0.01). Metformin was able to reduce senescence by upregulating the expression of RNA-binding protein MBNL1 and miR-130a-3p and reducing STAT3 expression. MBNL1 prolonged the half-life of miR-130a-3p, and miR-130a-3p could negatively regulate STAT3 by binding to its mRNA 3′UTR. In db/db diabetic mice, we found an enhanced senescence level combined with low expression of MBNL1 and miR-130a-3p and high expression of STAT3 compared with db/m control mice during nephropathy development. Meanwhile, metformin (200 mg/kg/day) could increase the expression of MBNL1 and miR-130a-3p and decreased STAT3 expression, thus reducing this senescence in db/db mice. Our results suggest that metformin reduces the senescence of renal tubular epithelial cells in diabetic nephropathy via the MBNL1/miR-130a-3p/STAT3 pathway, which provided new ideas for the therapy of this disease.

## 1. Introduction

Diabetes is a metabolic disorder characterized by elevated blood glucose levels [1]. The increasing morbidity of diabetes exposes more patients to diabetic complications, e.g., diabetic nephropathy [2], which is the major contributor to end-stage renal disease (ESRD) and involves renal glomerular, vascular, and tubular injuries [3, 4]. Studies have revealed that renal tubular epithelial cells present premature senescence in type II diabetic nephropathy, indicating that senescence of renal tubular epithelial cells is one of the mechanisms involved in the progression of diabetic nephropathy [5]. The occurrence and development of various diseases can trigger cell senescence, and the aged cells can drive and accelerate disease progression [6]. That is, the senescence program is implicated in diverse biological processes. For example, senescence can cause microvascular lesions in type II diabetes [7]. The high-glucose-induced accelerated senescence of renal tubular epithelial cells is an important cellular event that precedes renal interstitial injury in diabetic nephropathy [8].

Metformin is a biguanide derivative and a first-line oral therapeutic drug for type II diabetes [2]. Metformin has several hypoglycemic effects, for example, by inhibiting glucose absorption, enhancing peripheral insulin sensitivity, reducing glucose synthesis, and improving glucose availability [9, 10]. As previously shown, metformin can decrease both the blood glucose levels, as well as partially reversing the renal damage caused by diabetic nephropathy and prolonging the survival of diabetic mice [11, 12].

RNA-binding proteins (RBPs) can directly bind to RNA, thus forming a ribonucleoprotein complex, and in this way, they regulate the biological functions of RNA [13]. Studies have shown that RBPs are associated with diabetic nephropathy and senescence. Sheng et al. found that heterogeneous nuclear ribonucleoprotein F (hnRNP F) ameliorated interstitial fibrosis of renal tubules in the diabetic nephropathy mice [14]. Similarly, heterogeneous nuclear ribonucleoprotein A1 (hnRNP A1) could inhibit the senescence of human lung fibroblasts by upregulating SIRT1 expression [15]. In addition, MBNL1 is an RBP consisting of 343 amino acids and located at chromosome 3q25.1-q25.2, and its location imbalance in cells is an important pathogenic factor for myotonic dystrophy [16]. MBNL1 can bind to several RNAs to regulate their functions including stability [17]. It can bind to two tumor suppressors drebrin-like protein (DBNL) and transforming acidic coiled-coil containing protein 1 (TACC1) to maintain their stability and thus inhibit the invasion and metastasis of breast cancer [18]. More importantly, Lee et al. explored the influence of MBNL1 on the life of mice and found that MBNL1-knockout mice had significantly shorter lives [19]. However, there are currently no reports about the effects of metformin or MBNL1 on diabetic nephropathy-associated senescence.

miRNAs are noncoding RNAs with conservative sequences and composed of 21-25 nucleotides; miRNAs inhibit the expression of target genes by binding with the corresponding mRNA 3′UTR, thus regulating several cellular biological activities including cell differentiation, proliferation, apoptosis, and migration [20]. Some studies have suggested that miRNAs play an important role in hypertension caused by diabetic nephropathy [21], and the key enzyme Dicer—produced by miRNA knockout—can induce the progressive injuries of renal glomeruli and tubules [22]. Liu et al. observed that miR-25 could reverse the progression of diabetic nephropathy in mice [23]. Furthermore, Wu et al. found that miR-455-3p could improve glomerular hypertrophy, mesenchymal hyperplasia, and renal fibrosis of mice with diabetic nephropathy [24]. miR-130a-3p is located at chromosome 11q12.a, and it has been demonstrated that miR-130a-3p expression was reduced in the blood and liver of elderly mice [25]. By analysis and prediction using RBPDB software in this study, we found that MBNL1 and miR-130a-3p are potential binding partners.

STAT3 (signal transmission and transcription activation factor 3) is located at chromosome 17 q21.2 and can act on polypeptide receptors on the cell surface, resulting in the activation of various biological pathways [26]. STAT3 plays an important role in the pathogenesis of diabetic nephropathy [27, 28]. Waters et al. found that the stress-induced senescence of lung fibroblasts was related to STAT3 activation [29]. Moreover, in the senescence process of hepatic stellate cells, the STAT3/p53/p21 signaling pathway was shown to be activated [30]. A potential binding site of miR-130a-3p and STAT3 was predicted with targetScan and miRanda software in our study, but the action mechanism of miR-130a-3p and STAT3 has not yet been reported.

This study is aimed at providing a new theoretical and experimental basis for the pathogenesis of diabetic nephropathy and the relevance of treatment with metformin for this disease. We firstly explored the endogenous expression of MBNL1, miR-130a-3p, and STAT3 in diabetic nephropathy and their relation with the senescence of renal tubular epithelial cells. We then determined the regulation patterns among MBNL1, miR-130a-3p, and STAT3 and further investigated whether metformin was able to protect renal tubular epithelial cells from senescence via the MBNL1/miR-130a-3p/STAT3 pathway.

## 2. Materials and Methods

### 2.1. Cell Culture

The HK-2 human renal proximal tubular epithelial cell line was purchased from the Shanghai Institute for Biological Sciences Cell Resource Center. The HK-2 cells were cultured in normal glucose (NG) Dulbecco's Modified Eagle Medium/Nutrient Mixture F-12 (DMEM/F-12) that was supplemented with 10% fetal bovine serum (FBS; ExCell, FSP500). Normal glucose DMEM/F-12 was a 1 : 1 mixture of DMEM (Gibco, catalog no. 11966025) and Ham's F-12 (Gibco, catalog no. 11765054) that contained 5.56 mmol/L D-glucose (Sigma-Aldrich, catalog no. 47829). The cells were maintained at 37°C in a humidified 5% CO_2_ incubator. To induce cell senescence, HK-2 cells were treated with high-glucose DMEM/F-12 that contained 30 mmol/L D-glucose for 72 h. For treatment with metformin (Selleck, catalog no. S1950), HK-2 cells were pretreated with metformin (1 mmol/L) for 2 h after serum starvation for 24 h and then incubated with normal or high-glucose DMEM/F12 for 72 h, for treatment with A-769662 (Selleck, catalog no. S2697) and Dorsomorphin (Selleck, catalog no. S7840).

### 2.2. Cell Transfection

Short-hairpin MBNL (sh-MBNL1) was constructed in a GV493 lentiviral vector, and sh-STAT3 was constructed in a GV248 lentiviral vector. Full-length MBNL1 and STAT3 genes were constructed in a GV492 lentiviral vector. All of the lentivirus and their respective negative controls were synthesized (Genechem, Shanghai, China). The miR-130a-3p mimics, miR-130a-3p inhibitor, and their respective negative controls were synthesized (GenePharma, Shanghai, China). The cells were seeded in 24-well plates (Corning), and transfection was performed until 80% confluency was reached. For lentivirus transfection, HitransG A (Genechem, catalog no. REVG003) was used according to the manufacturer's instructions to transfect cells with the lentivirus. The stable cell lines were selected using puromycin screening. For miRNA transfection, the cells were transfected with the miR-130a-3p mimics, miR-130a-3p inhibitor, or their respective negative controls using Lipofectamine 2000 Reagent (Invitrogen, catalog no. 11668019). The efficiency of transfection was analyzed using quantitative real-time polymerase chain reaction (qRT-PCR) or Western blot. The sequences for shRNAs and RNA oligoribonucleotides were as follows: sh-MBNL1 (5′-GCCAACCAGATACCCATAATA-3′), sh-STAT3 (5′-CGGCGTCCAGTTCACTACTAA-3′), and miR-130a-3p inhibitor (5′-AUGCCCUUUUAACAUUGCACUG-3′).

### 2.3. Mouse Model of Diabetic Nephropathy

Thirty male db/db mice (8 weeks old) and 15 male db/m control mice (8 weeks old) were purchased from the Model Animal Research Center of Nanjing University. All the mice were housed in a temperature-controlled room (21°C ± 1°C) under a 12 h/12 h light/dark cycle. The db/db mice were randomly selected and divided into two groups (in accordance with the random number table): db/db group and db/db+metformin treatment (db/db+Met) group (*n* = 15/group). Five mice from each group were sacrificed (isoflurane anesthesia followed by cervical dislocation, similarly hereinafter) at that time (when the mice were 8 weeks old). The db/db+Met group was then injected intraperitoneally with metformin (200 mg/kg/day, once daily, dissolved in saline), and the db/m group and db/db group were injected intraperitoneally with vehicle (saline). Eight weeks later, when the mice were 16 weeks old, another five mice in each group were sacrificed. Finally, the remaining five mice in each group were sacrificed 16 weeks after treatment when the mice were 24 weeks old. All experimental procedures involving animals were done in accordance with the Guide for the Care and Use of Laboratory Animals (NIH publication no. 80-23, revised 1996) and the institutional ethical guidelines for animal experiments. The ethical approval was acquired from the Research Ethics Committee of the First Affiliated Hospital of China Medical University.

### 2.4. Groups

To determine the effect of high glucose on HK-2 cell senescence, the cells were divided into three groups: normal glucose (NG; 5.56 mmol/L D-glucose), mannitol (MA; 5.56 mmol/L D-glucose plus 24.44 mmol/L mannitol), and high glucose (HG; 30 mmol/L D-glucose). To determine the effect of MBNL1, the following groups were formed: MBNL1(+) (transfected with MBNL1 overexpression plasmid), MBNL1(-) (transfected with sh-MBNL1 plasmid), MBNL1(+)-NC (transfected with negative control (NC) plasmid for MBNL1), and MBNL1(-)-NC (transfected with sh-NC plasmid for sh-MBNL1 plasmid). To determine the effect of miR-130a-3p, the following groups were formed: miR-130a-3p(+) (transfected with miR-130a-3p mimics), miR-130a-3p(-) (transfected with miR-130a-3p inhibitor), miR-130a-3p(+)-NC (transfected with NC mimics), and miR-130a-3p(-)-NC (transfected with NC inhibitor). To determine the effect of STAT3, the following groups were formed: STAT3(+) (transfected with STAT3 overexpression plasmid), STAT3(-) (transfected with sh-STAT3 plasmid), STAT3(+)-NC (transfected with NC plasmid for STAT3), and STAT3(-)-NC (transfected with sh-NC plasmid for sh-STAT3 plasmid).

### 2.5. qRT-PCR

Total RNA was extracted from HK-2 cells using TRIzol reagent (Life Technologies, catalog no. 15596026). The concentration and quality of RNA were determined using a Nanodrop Spectrophotometer (ND-100) at a 260/280 nm ratio. Reverse transcription was performed using the riboSCRIPT Reverse Transcription Kit (RiboBio, catalog no. C11027-2) or PrimeScript RT reagent Kit with gDNA Eraser (Takara, catalog no. RR047A). The expression levels of miR-130a-3p and U6 were determined using the Bulge-Loop miRNA qRT-PCR Starter Kit (RiboBio, catalog no. C10211-2), with U6 as the internal control. The expression levels of MBNL1, STAT3, and GAPDH were determined using TB Green Premix Ex Taq II (Takara, catalog no. RR820A), with GAPDH as the internal control. Real-time PCR was performed using the ABI 7500 Fast Real-Time PCR System (Applied Biosystems). The 2^−*ΔΔ*Ct^ relative quantification method was used to calculate RNA expression. The primers for miR-130a-3p and U6 were synthesized by RiboBio. The following primers for MBNL1, STAT3, and GAPDH were synthesized by Sangon Biotech: MBNL1 (forward, 5′-ATGGCTGTTAGTGTCACACCA-3′; reverse, 5′-CATGTTCTTCTGCTGAATCAA-3′), STAT3 (forward, 5′-AGAAGGACATCAGCGGTAAG-3′; reverse, 5′-CCTTGGGAATGTCAGGATAGAG-3′), and GAPDH (forward, 5′-CGGATTTGGTCGTATTGGG-3′; reverse, 5′-CTGGAAGATGGTGATGGGATT-3′).

### 2.6. Western Blot

Total protein was extracted from HK-2 cells using RIPA lysis buffer (Beyotime, catalog no. P0013K) for 30 min on ice. The samples were centrifuged at 10,000 g at 4°C for 10 min. Protein concentrations were then determined using the BCA protein assay kit (Beyotime, catalog no. P0012). Proteins were separated by sodium dodecyl sulfate-polyacrylamide gel electrophoresis (SDS-PAGE) using 10% acrylamide resolving gel and transferred to polyvinylidene difluoride (PVDF) membranes. The PVDF membranes were then incubated in TBST (0.1% Tween-20) that contained 5% nonfat milk for 2 h at room temperature, followed by incubation for 18 h with primary antibodies. The membranes were subsequently incubated with the appropriate corresponding horseradish peroxidase-conjugated secondary antibody (Proteintech, catalog no. SA00001-1 or SA00001-2) for 2 h. Immunoblots were visualized by enhanced chemiluminescence (Millipore, catalog no. WBKLS0100) and scanned using ChemImager 5,500 v.2.03 software. The relative integrated density values (IDVs) were calculated using ImageJ software based on GAPDH as the internal control. The primary antibodies were as follows: MBNL1 (Santa Cruz Biotechnology, catalog no. sc-47740), STAT3 (Proteintech, catalog no. 10253-2-AP), p21 (Cell Signaling Technology, catalog no. 2947), GAPDH (Proteintech, catalog no. 60004-1-Ig), P-AMPK (Cell Signaling Technology, catalog no. 2535S), and AMPK (Proteintech, catalog no. 10929-2-AP). The unedited blots in all the figures are provided in Supplementary Materials ([Supplementary-material supplementary-material-1]).

### 2.7. SA-*β*-gal Staining

HK-2 cells were cultured in six-well plates, and tissue sections were deparaffinized and hydrated. For *β*-galactosidase staining (Cell Signaling Technology, catalog no. 9860) of cell senescence, cells or sections were rinsed once with phosphate-buffered saline (PBS) and fixed with 1× fixative solution for 15 min at room temperature. The samples were rinsed twice with PBS and incubated with *β*-galactosidase staining solution overnight at 37°C in a dry incubator (without CO_2_). An Olympus DP71 microscope (Olympus, Tokyo, Japan) was used to acquire photomicrographs. For the cell experiments, blue-stained cells were counted in five randomly chosen fields per well to determine the percentage of SA-*β*-gal-positive cells (200x magnification). For tissue sections, the ratio of the blue-stained area was evaluated relative to the total area in five randomly chosen renal cortical areas per section (200x magnification). The percentages of SA-*β*-gal-positive cells or areas were averaged and quantified for the statistical analysis.

### 2.8. Nascent RNA Capture

The Cell-Light EU Apollo567 RNA Stain Kit (RiboBio, catalog no. C10316-1) was used to detect nascent RNA according to the manufacturer's protocol. HK-2 cells were treated with ethynyl uridine (EU), in which EU-nascent RNA binds to fluorochrome, which can be fluorescently detected. An Olympus BX51 fluorescence microscope (Olympus, Tokyo, Japan) was used to acquire the photomicrographs.

### 2.9. RNA Half-Life Assay

After transfection with sh-NC or sh-MBNL1, HK-2 cells were treated with 5 *μ*g/mL actinomycin D. Total RNA was collected at different time points and quantified using qRT-PCR.

### 2.10. Reporter Vector Constructs and Luciferase Reporter Assay

The potential binding site of miR-130a-3p in the STAT3 3′-untranslated region sequence was amplified by PCR and cloned into a pmirGlo Dual-luciferase miRNA Target Expression Vector (Promega, Madison, WI) to construct the luciferase reporter vector (STAT3-Wt; GenePharma). The sequence of the putative miR-130a-3p binding site was replaced to generate STAT3-Mut. HK-2 cells were cotransfected with STAT3-Wt (or STAT3-Mut) plasmids and miR-130a-3p mimics (or miR-130a-3p-NC). After transfection using the Dual-Luciferase reporter assay kit (Promega) for 48 h, luciferase activity was measured. HK-2 cells were divided into five groups: control, STAT3-Wt+miR-130a-3p-NC (transfected with STAT3-Wt and miR-130a-3p-NC), STAT3-Wt+miR-130a-3p (transfected with STAT3-Wt and miR-130a-3p), STAT3-Mut+miR-130a-3p-NC (transfected with STAT3-Mut and miR-130a-3p-NC), and STAT3 Mut+miR-130a-3p8 (transfected with STAT3-Mut and miR-130a-3p).

### 2.11. RNA Immunoprecipitation Assay

The RNA-binding immunoprecipitation (RIP) assay was performed using the Imprint RNA Immunoprecipitation Kit (Sigma-Aldrich, catalog no. RIP-12RXN) according to the manufacturer's protocol. The HK-2 cell lysate was incubated with RIP buffer that contained magnetic beads that were conjugated with MBNL1 antibody or immunoglobulin G (IgG) from mouse serum as a negative control. The magnetic beads were washed, and RBP-binding RNA was purified. The RNA concentration was measured using a NanoDrop spectrophotometer, and the RNA samples were analyzed using qRT-PCR.

### 2.12. RNA Pull-Down Assay

The Pierce Magnetic RNA-Protein Pull-Down Kit (Thermo Fisher Scientific, catalog no. 20164) was used to detect the interaction between MBNL1 and miR-130a-3p according to the manufacturer's protocols. miR-130a-3p transcripts were transcribed using the MEGAshortscript T7 Transcription Kit (Thermo Fisher Scientific, catalog no. AM1354). The Pierce RNA 3′ End Desthiobiotinylation Kit (Thermo Fisher Scientific, catalog no. 20163) was used to biotin-label the RNAs. Biotin-labeled miR-130a-3p or antisense RNA was incubated with magnetic beads and HK-2 cell lysates. The RNA-binding protein complexes were then washed and eluted from the magnetic beads and detected by Western blot.

### 2.13. Immunohistochemistry

The kidneys were fixed in 4% paraformaldehyde for immunohistochemical staining. Sections (4 *μ*m) were deparaffinized in xylene and hydrated in graded alcohol and water. Antigen was retrieved with a citrate-based solution. The sections were incubated with 3% H_2_O_2_ in methanol to block endogenous peroxidase activity. Sections were blocked with goat serum or 3% bovine serum albumin (BSA) for 30 min at room temperature and then incubated with MBNL1 (Santa Cruz Biotechnology, catalog no. sc-47740) and STAT3 (Cell Signaling Technology, catalog no. 30835) overnight at 4°C. After three washes in PBS, the sections were incubated with anti-rabbit or anti-mouse secondary antibody for 2 h at room temperature and subjected to 3,3′-diaminobenzidine (DAB) to elicit a brown color. Finally, the sections were counterstained with hematoxylin for 3 min. Images were acquired using an optical microscope. The final image analysis was performed using ImagePro Plus 6.0 software.

### 2.14. In Situ Hybridization

Dig-labeled probes were designed and synthesized (Servicebio, Wuhan, China). The kidneys were fixed in 4% paraformaldehyde, and 4 *μ*m sections were deparaffinized in xylene and hydrated in a graded series of alcohol. The sections were treated with 20 *μ*g/mL proteinase K for 30 min at 37°C. The sections were pretreated with hybridization buffer at 37°C for 1 h and then hybridized with an mmu-miR-130a-3p probe at 37°C overnight. The sections were washed in 2 × SSC for 10 min at 37°C, followed by two 5 min washes in 1 × SSC at 37°C and then a 10 min wash in 0.5 × SSC at room temperature. The sections were blocked with BSA for 30 min at room temperature. After incubating with anti-DIG-HRP for 40 min at 37°C, the sections were subjected to DAB to elicit a brown color. Finally, the sections were counterstained with hematoxylin for 3 min. Images were acquired using an optical microscope.

### 2.15. Statistical Analysis

The data are expressed as the mean ± SD. All statistical analyses in this study were performed using SPSS 20.0 software (IBM, New York, NY, USA). As the variances between groups are similar and the data distributed normality, Student's *t*-test was used to analysis the difference between two groups. One-way ANOVA with Bonferroni's method was used to determine the difference among multiple groups. Values of *p* < 0.05 were considered statistically significant.

## 3. Results

### 3.1. Metformin Reduced the High-Glucose-Induced Senescence of Renal Tubular Epithelial Cells

**Figure 1:**
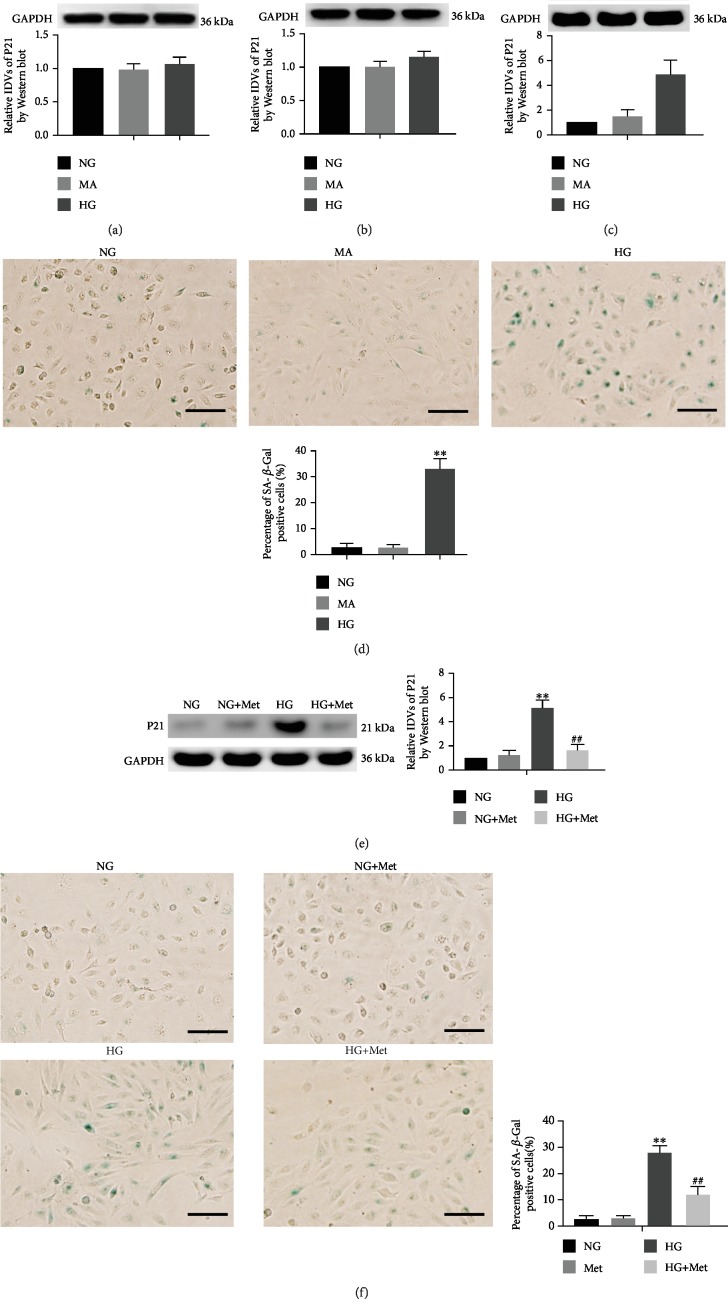
Metformin reduced HK-2 cell senescence that was induced by HG. (a–c) Western blot was used to determine the expression of P21 in HK-2 cells that were treated with NG, MA, and HG at different times. (d) The percentage of SA-*β*-gal-positive cells was detected in HK-2 cells that were exposed to NG, MA, and HG for 72 h. (e) P21 protein expression levels and (f) the percentage of SA-*β*-gal-positive cells were detected in HK-2 cells that were exposed to NG, NG+Met, HG, and HG+Met for 72 h. The data are expressed as the mean ± SD (*n* = 3/group). ^∗∗^*p* < 0.01, vs. the NG group; ^##^*p* < 0.01, vs. HG group. Scale bars = 50 *μ*m.

After high-glucose treatment for 24, 48, and 72 h, the expressions of senescence-associated protein P21 in renal tubular epithelial (HK-2) cells were detected by Western blot. At 24 and 48 h, there was no statistically significant difference between the high-glucose (HG) group and the mannitol hypertonic control (MA) group or the normal glucose (NG) group on P21 expression (Figures 1(a) and 1(b)). At 72 h, the expression of P21 was significantly higher in the HG group than in the NG group, but there was no statistical difference between the MA group and the NG group (Figure 1(c)). The subsequent HG group was performed under high-glucose condition for 72 h.

Senescence of HK-2 cells was detected using the *β*-galactosidase staining method. The *β*-galactosidase positive staining rate was significantly higher in the HG group than in the NG group, but there was no statistical difference between the MA group and the NG group (Figure 1(d)). Furthermore, P21 expression and *β*-galactosidase activity were significantly lower in the metformin (Met) treatment (HG+Met) group than in the HG group (Figures 1(e) and 1(f)).

### 3.2. Metformin Reduced the High-Glucose-Induced Senescence of Renal Tubular Epithelial Cells by Upregulating MBNL1

**Figure 2:**
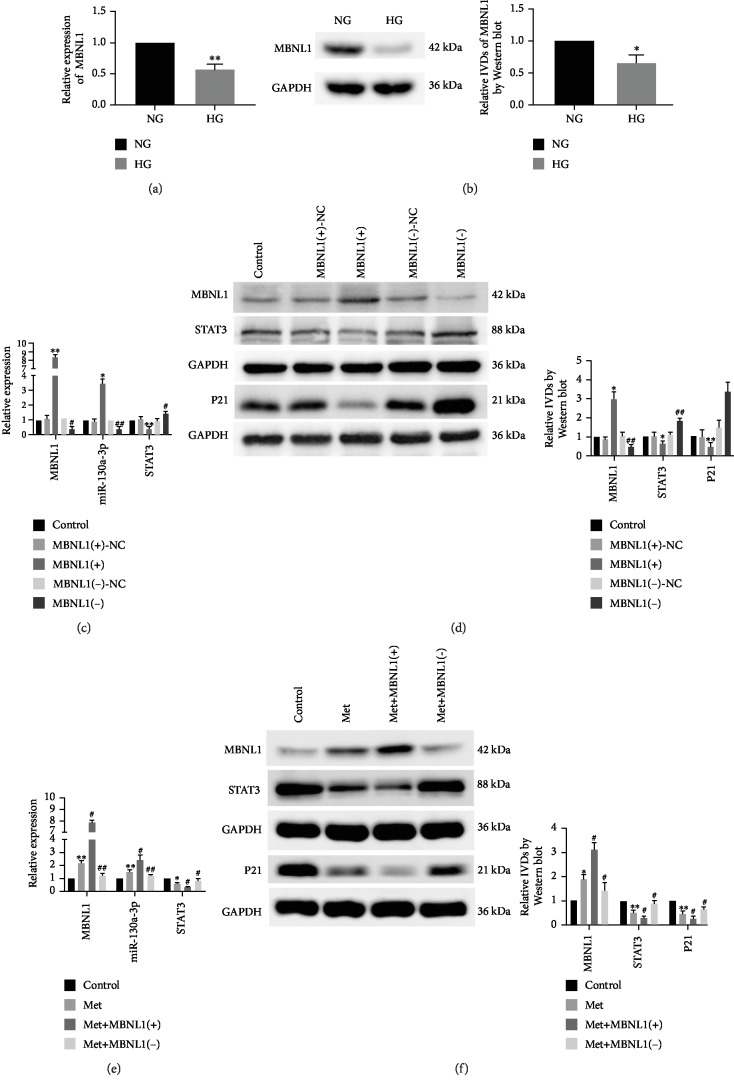
MBNL1 was downregulated in senescent HK-2 cells, and metformin reduced cell senescence in HK-2 cells by upregulating MBNL1. (a) qRT-PCR was performed to detect expression levels of MBNL1 in HK-2 cells that were cultured in NG and HG for 72 h. (b) MBNL1 protein expression levels in HK-2 cells that were cultured in NG and HG for 72 h. The data are expressed as the mean ± SD (*n* = 3/group). ^∗^*p* < 0.05, *vs*. the NG group; ^∗∗^*p* < 0.01, *vs*. the NG group. (c) The expressions of MBNL1, miR-130a-3p, and STAT3 were detected in HK-2 cells with MBNL1 overexpression or inhibition by qRT-PCR. (d) The protein expression levels of MBNL1, STAT3, and P21 were detected to determine the effect of MBNL1 on senescence in HK-2 cells. The data are expressed as the mean ± SD (*n* = 5/group). ^∗^*p* < 0.05, *vs*. the MBNL1(+)-NC group; ^∗∗^*p* < 0.01, *vs*. the MBNL1(+)-NC group; ^#^*p* < 0.05, *vs*. the MBNL1(-)-NC group; ^##^*p* < 0.01, *vs*. MBNL1(-)-NC group. (e) The expressions of MBNL1, miR-130a-3p, and STAT3 were detected in HK-2 cells treated with metformin by qRT-PCR. (f) The protein expression levels of MBNL1, STAT3, and P21 were detected in HK-2 cells that were treated with metformin. The data are expressed as the mean ± SD (*n* = 3/group). ^∗^*p* < 0.05, *vs*. the control group; ^∗∗^*p* < 0.01, *vs*. the control group; ^#^*p* < 0.05, *vs*. the Met group; ^##^*p* < 0.01, *vs*. the Met group.

We firstly investigated the effect of high glucose on MBNL1 and found that mRNA and protein expression levels of MBNL1 in the HG group (Figures 2(a) and 2(b)) were significantly lower compared with those in the NG group. Subsequent investigations in this part were performed under HG conditions. We then studied the effects of overexpressed or silenced MBNL1 on the senescence. Compared with the MBNL1(+)-NC group, MBNL1 mRNA and protein expression levels and the miR-130a-3p expression level were significantly increased, but STAT3 mRNA and protein expression levels and the protein expression level of P21 were markedly decreased in the MBNL1(+) group. Conversely, compared with the MBNL1(-)-NC group, MBNL1 mRNA and protein expression levels and miR-130a-3p expression level were significantly decreased, but STAT3 mRNA and protein expression levels and the protein expression level of P21 were markedly increased in the MBNL1(-) group (Figures 2(c) and 2(d)). To further explore whether metformin reduced the senescence of HK-2 cells via MBNL1, metformin was used in combination with MBNL1-overexpressed or MBNL1-silenced stably transfected HK-2 cells. Results showed that compared with the control group, MBNL1 mRNA and protein expression levels and miR-130a-3p expression were significantly increased, but STAT3 mRNA and protein expression levels and the protein expression level of P21 were evidently decreased in the Met group; the same changes were demonstrated in the Met+MBNL1(+) group, but the opposite changes were evident in the Met+MBNL1(-) group, compared with the Met group. This indicated that silencing of MBNL1 could reverse the inhibition of metformin on the senescence of renal tubular epithelial cells (Figures 2(e) and 2(f)).

### 3.3. Metformin Reduced the High-Glucose-Induced Senescence of Renal Tubular Epithelial Cells by Upregulating miR-130a-3p

**Figure 3:**
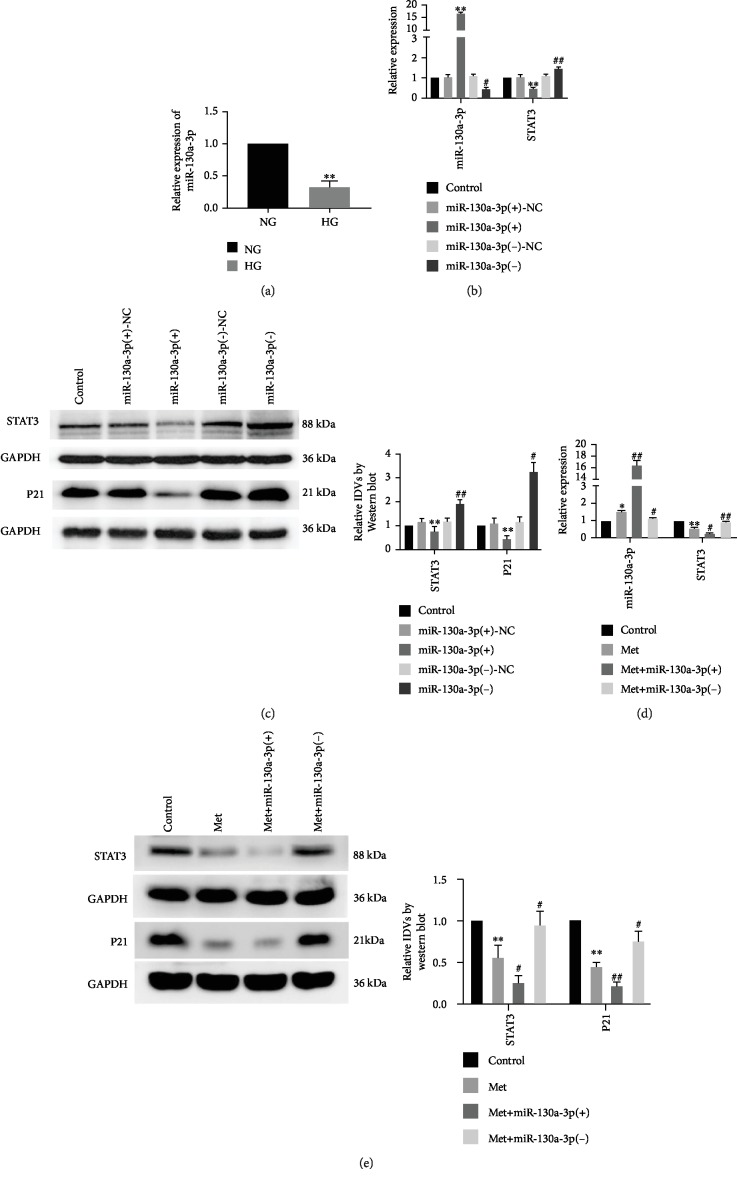
miR-130a-3p was downregulated in senescent HK-2 cells, and metformin reduced cell senescence in HK-2 cells by upregulating miR-130a-3p. (a) qRT-PCR was used to detect expression levels of miR-130a-3p in HK-2 cells that were cultured in NG and HG for 72 h. The data are expressed as the mean ± SD (*n* = 3/group). ^∗∗^*p* < 0.01, *vs*. NG group. (b) The RNA expression levels of miR-130a-3p and STAT3 were detected in HK-2 cells after miR-130a-3p overexpression or knockdown. (c) The protein expression levels of STAT3 and P21 were detected in HK-2 cells. The data are expressed as the mean ± SD (*n* = 5/group). ^∗∗^*p* < 0.01, *vs*. the miR-130a-3p(+)-NC group; ^#^*p* < 0.05, *vs*. the miR-130a-3p(-)-NC group; ^##^*p* < 0.01, *vs*. the miR-130a-3p(-)-NC group. (d) The RNA expression levels of miR-130a-3p and STAT3 were detected in HK-2 cells after treatment with metformin. (e) The protein expression levels of STAT3 and P21 were detected in HK-2 cells after treatment with metformin. The data are expressed as the mean ± SD (*n* = 3/group). ^∗^*p* < 0.05, *vs*. the control group; ^∗∗^*p* < 0.01, *vs*. the control group; ^#^*p* < 0.05, *vs*. the Met group; ^##^*p* < 0.01, *vs*. the Met group.

As shown by the detection results concerning the regulation of high glucose by miR-130a-3p in this study, miR-130a-3p expression was significantly decreased in the HG group, compared with the NG group (Figure 3(a)). Subsequent investigations in this part were performed under HG conditions. The effects of overexpressed or silenced miR-130a-3p on the senescence were investigated. Compared with the miR-130a-3p(+)-NC group, the miR-130a-3p expression level was significantly increased, but STAT3 mRNA and protein expression levels and the protein expression level of P21 were markedly decreased in the miR-130a-3p(+) group. Conversely, compared with the miR-130a-3p(-)-NC group, the miR-130a-3p expression level was significantly decreased, but STAT3 mRNA and protein expression levels and the protein expression level of P21 were markedly increased in the miR-130a-3p(-) group (Figures 3(b) and 3(c)). To explore whether metformin acted via miR-130a-3p, metformin was used in combination with miR-130a-3p-overexpressed or miR-130a-3p-silenced stably transfected HK-2 cells. The miR-130a-3p expression was significantly increased, but STAT3 mRNA and protein expression levels and the protein expression level of P21 were significantly decreased in the Met group compared with the control group; the same changes were demonstrated in the Met+miR-130a-3p(+) group, but the opposite changes were evident in the Met+miR-130a-3p(-) group compared with the Met group. This suggests that silencing of miR-130a-3p expression could reverse the inhibition of metformin on the senescence of renal tubular epithelial cells (Figures 3(d) and 3(e)).

### 3.4. Metformin Increased the Stability of miR-130a-3p by Upregulating MBNL1 and Thus Reduced the High-Glucose-Induced Senescence of Renal Tubular Epithelial Cells

**Figure 4:**
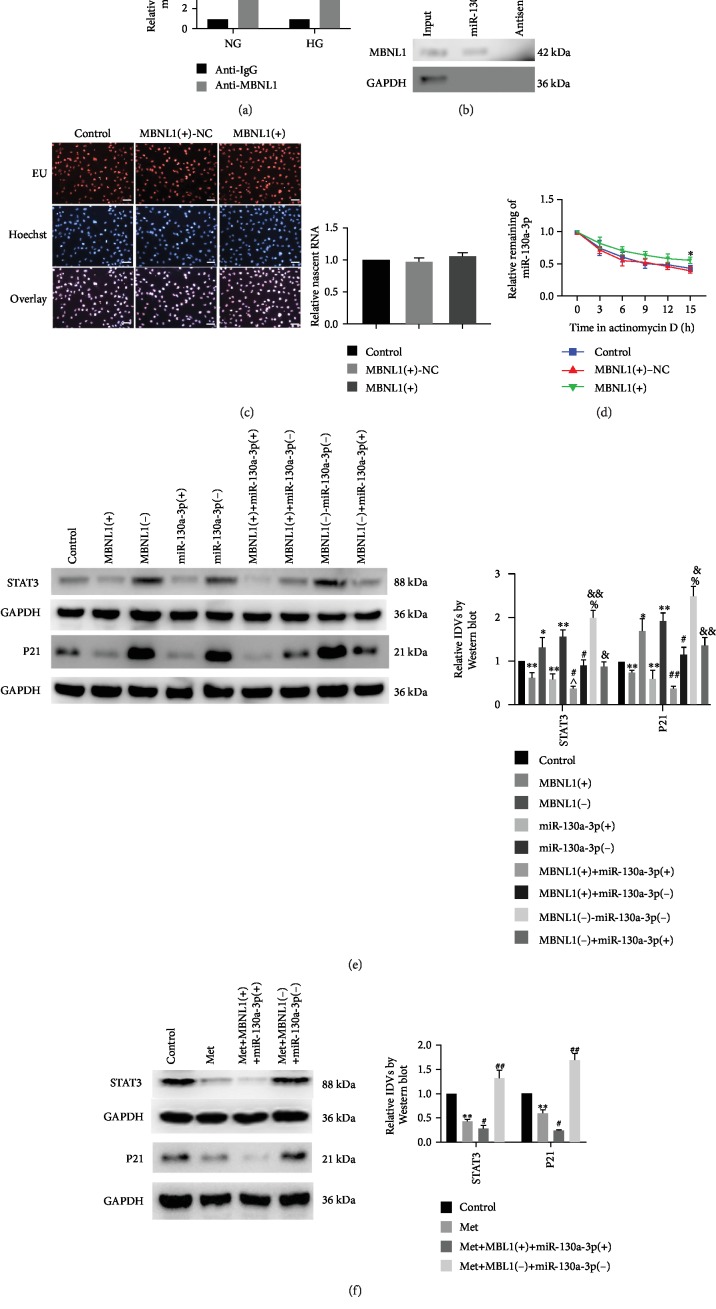
MBNL1 bound with miR-130a-3p, and metformin reduced cell senescence in HK-2 cells by upregulating MBNL1/miR-130a-3p signaling. (a) miR-130a-3p was identified in the MBNL1 complex. miR-130a-3p enrichment was measured using qRT-PCR. The data are expressed as the mean ± SD (*n* = 3/group). ^∗^*p* < 0.05, *vs*. the anti-IgG group; ^∗∗^*p* < 0.01, *vs*. the anti-IgG group; ^##^*p* < 0.01, *vs*. the NG group. (b) The levels of MBNL1 and GAPDH proteins that immunoprecipitated with miR-130a-3p were evaluated by Western blot. The expression levels of MBNL1 and GAPDH proteins are shown. (c) The Cell-Light EU Apollo567 RNA Stain Kit was used to label and capture newly synthesized RNA. (d) Relative levels of miR-130a-3p at different actinomycin D treatment times in the control group, MBNL1(+)-NC group, and MBNL1(+) group. (e) The expression levels of STAT3 and P21 were detected to determine the effect of MBNL1 and miR-130a-3p on senescence in HK-2 cells. The data are expressed as the mean ± SD (*n* = 3/group). ^∗^*p* < 0.05, *vs*. the control group; ^∗∗^*p* < 0.01, *vs*. the control group; ^#^*p* < 0.05, *vs*. the MBNL1(+) group; ^##^*p* < 0.01, *vs*. the MBNL1(+) group; ^&^*p* < 0.05, *vs*. the MBNL1(-) group; ^&&^*p* < 0.01, *vs*. the MBNL1(-) group; ^∧^*p* < 0.05, *vs*. the miR-130a-3p(+) group; ^∧∧^*p* < 0.01, *vs*. the miR-130a-3p(+) group; ^%^*p* < 0.05, *vs*. the miR-130a-3p(-) group. (f) STAT3 and P21 protein expression levels were detected in HK-2 cells after treatment with metformin. The data are expressed as the mean ± SD (*n* = 3/group). ^∗∗^*p* < 0.01, *vs*. the control group; ^#^*p* < 0.05, *vs*. the Met group; ^##^*p* < 0.01, *vs*. the Met group.

A potential binding site of MBNL1 for miR-130a-3p was predicted by analysis with RBPDB software. RNA-binding protein immunoprecipitation (RIP) results showed that miR-130a-3p was significantly more enriched by anti-MBNL1 compared with the anti-IgG in NG group; a similar change was detected in the HG group. Moreover, the enrichment of miR-130a-3p by anti-MBNL1 was significantly lower in the HG group than in the NG group (Figure 4(a)). Furthermore, RNA pull-down assay results indicated that MBNL1 bound to miR-130a-3p (Figure 4(b)). Subsequent investigations in this part were performed under HG conditions. EU nascent RNA detection results showed no statistical differences in RNA transcriptional activity between the control group and the MBNL1(+)-NC group or the MBNL1(+) group (Figure 4(c)). After treatment with actinomycin D, miR-130a-3p expression was detected, and the RNA half-life of miR-130a-3p in the MBNL1(+) group was significantly increased compared with that in the MBNL1(+)-NC group (Figure 4(d)). The effects on STAT3 expression and the senescence of renal tubular epithelial cells were detected by cotransfection of MBNL1 and miR-130a-3p. Results showed that the protein expression levels of STAT3 and P21 were significantly decreased in the MBNL1(+)+miR-130a-3p(+) group compared with the MBNL1(+) group or the miR-130a-3p(+) group and markedly increased in the MBNL1(+)+miR-130a-3p(-) group compared with the MBNL1(+) group. This suggests that silenced miR-130a-3p could reverse the inhibition on the senescence of renal tubular epithelial cells by MBNL1(+). The protein expression levels of STAT3 and P21 were significantly increased in the MBNL1(-)+miR-130a-3p(-) group compared with the MBNL1(-) group or the miR-130a-3p(-) group, but markedly decreased in the MBNL1(-)+miR-130a-3p(+) group compared with the MBNL1(-) group. This indicated that overexpressed miR-130a-3p could reverse the MBNL1(-)-induced senescence promoting effect on renal tubular epithelial cells (Figure 4(e)). These findings suggest that overexpressed MBNL1 reduced the high-glucose-induced senescence of renal tubular epithelial cells by specifically binding with miR-130a-3p and thus increasing its stability.

To explore whether metformin acted via the MBNL1/miR-130a-3p pathway, metformin was used to treat cells that had been transfected with MBNL1 and miR-130a-3p dual overexpression or dual silencing. As shown by the detection results concerning the effects on STAT3 expression and the senescence of renal tubular epithelial cells, the protein expression levels of STAT3 and P21 were significantly decreased in the Met group compared with the control group; the same changes were evident in the Met+MBNL1(+)+miR-130a-3p(+) group, but the opposite changes were shown in the Met+MBNL1(-)+miR-130a-3p(-) group compared with the Met group (Figure 4(f)).

### 3.5. Metformin Reduced the High-Glucose-Induced Senescence of Renal Tubular Epithelial Cells by Downregulating STAT3

**Figure 5:**
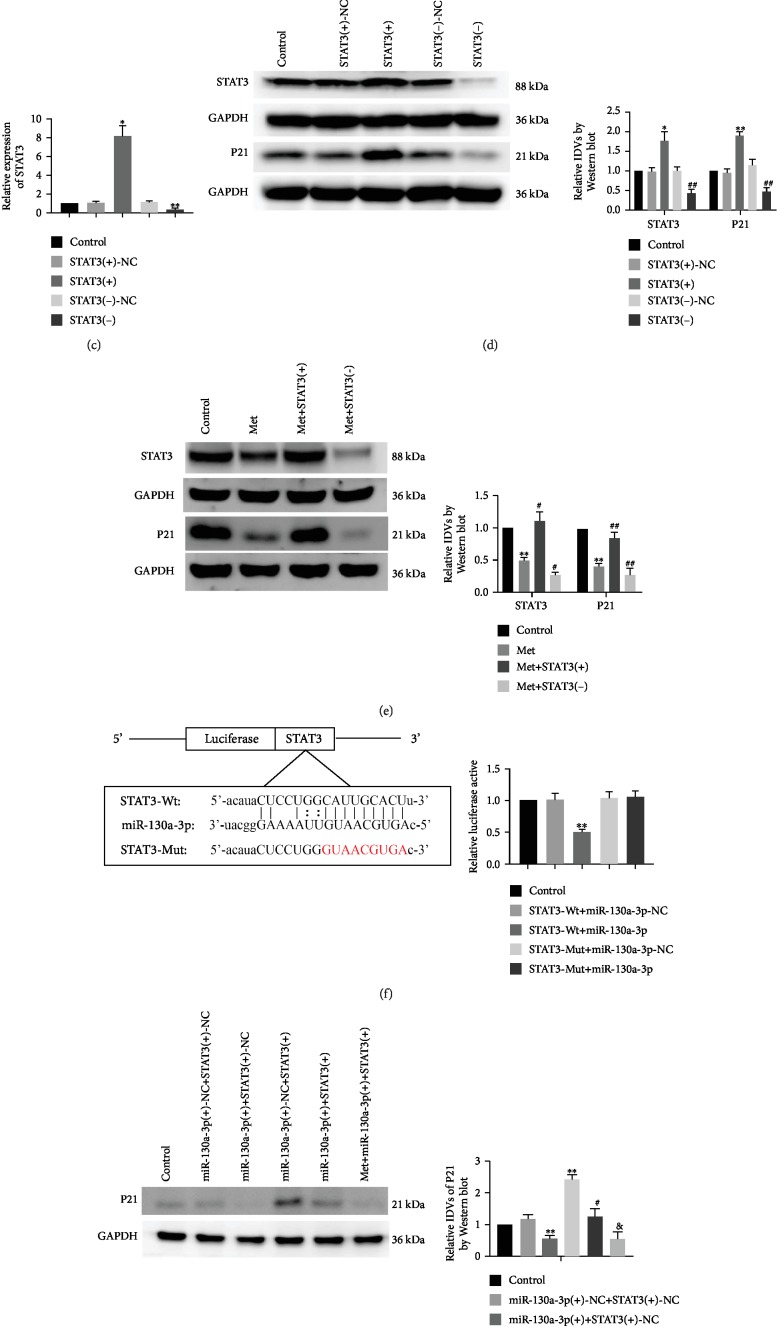
Metformin reduced cell senescence in HK-2 cells through miR-130a-3p/STAT3 signaling. (a) qRT-PCR was used to detect expression levels of STAT3 in HK-2 cells that were cultured in NG and HG for 72 h. (b) STAT3 protein expression levels in HK-2 cells that were cultured in NG and HG for 72 h. The data are expressed as the mean ± SD (*n* = 5/group). ^∗^*p* < 0.05, *vs*. the NG group; ^∗∗^*p* < 0.01, *vs*. the NG group. (c) The mRNA expression of STAT3 was detected in HK-2 cells. (d) The protein expressions of STAT3 and P21 were detected to determine the effect of STAT3 on senescence in HK-2 cells. The data are expressed as the mean ± SD (*n* = 3/group). ^∗^*p* < 0.05, *vs*. the STAT3(+)-NC group; ^∗∗^*p* < 0.01, *vs*. the STAT3(+)-NC group; ^##^*p* < 0.01, *vs*. the STAT3(-)-NC group. (e) The expressions of STAT3 and P21 were detected in HK-2 cells after treatment with metformin. The data are expressed as the mean ± SD (*n* = 3/group). ^∗∗^*p* < 0.01, *vs*. the control group; ^#^*p* < 0.05, *vs*. the Met group; ^##^*p* < 0.01, *vs*. Met the group. (f) Predicted miR-130a-3p binding site in STAT3 (STAT3-Wt) and mutant sequence (STAT3-Mut). The relative luciferase activity was conducted after cells were cotransfected with STAT3-WT (or STAT3-Mut) and miR-130a-3p-NC (or miR-130a-3p). The data are expressed as the mean ± SD (*n* = 3/group). ^∗∗^*p* < 0.01, *vs*. the STAT3-Wt+miR-130a-3p-NC group. (g) The expression of P21 was detected to determine the effect of miR-130a-3p, STAT3, and metformin on senescence in HK-2 cells. The data are expressed as the mean ± SD (*n* = 3/group). ^∗^*p* < 0.05, *vs*. the miR-130a-3p(+)-NC+STAT3(+)-NC group; ^∗∗^*p* < 0.01, *vs*. the miR-130a-3p(+)-NC+STAT3(+)-NC group; ^#^*p* < 0.05, *vs*. the miR-130a-3p(+)+STAT3(+)-NC group; ^&^*p* < 0.05, *vs*. the miR-130a-3p(+)+STAT3(+) group.

By investigating the effect of high glucose on STAT3 expression, it was found that the mRNA (Figure 5(a)) and protein (Figure 5(b)) levels of STAT3 in the HG group were significantly higher compared with the NG group. Subsequent investigations in this part were performed under HG conditions. Results concerning the effects of overexpressed or silenced STAT3 on the senescence of renal tubular epithelial cells showed that the STAT3 mRNA and protein expression levels and the protein expression of P21 were significantly increased in the STAT3(+) group compared with the STAT3(+)-NC group, but were markedly decreased in the STAT3(-) group compared with the STAT3(-)-NC group (Figures 5(c) and 5(d)). The effects on the senescence of renal tubular epithelial cells were detected after the combined use of metformin and STAT3-overexpressed or STAT3-silenced stably transfected cells. Results showed that the protein expression levels of STAT3 and P21 were significantly decreased in the Met group compared with the control group; the same changes were evident in the Met+STAT3(-) group, but opposite changes were found in the Met+STAT3(+) group compared with the Met group. This suggests that overexpressed STAT3 could reverse the metformin-induced inhibition on the senescence of renal tubular epithelial cells (Figure 5(e)).

**Figure 6:**
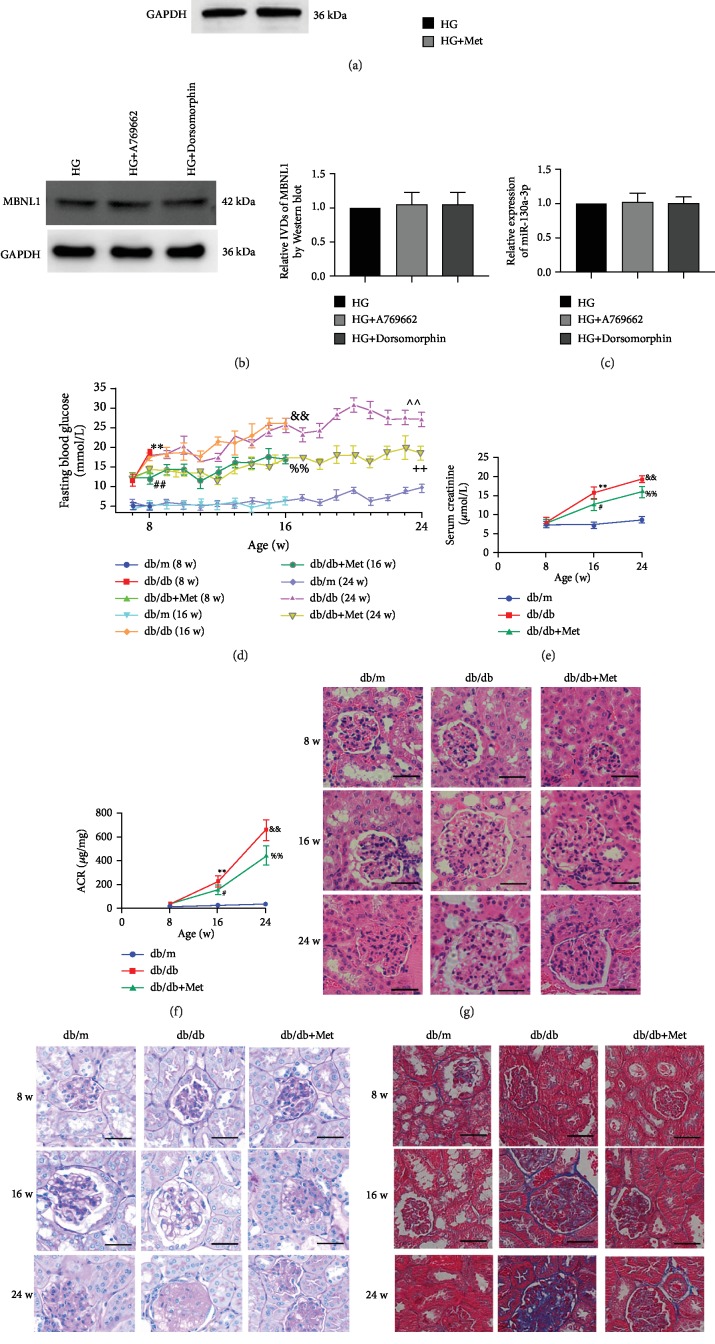
Metformin improved the renal function and pathological changes in db/db mice. (a) Western blot was used to determine the expressions of P-AMPK and AMPK in HK-2 cells that were treated with HG and HG+Met for 72 h. The data are expressed as the mean ± SD (*n* = 3/group). ^∗^*p* < 0.05, *vs*. the HG group; ^∗∗^*p* < 0.01, *vs*. the HG group. (b, c) The expressions of MBNL1 and miR-130a-3p were detected in HK-2 cells that were cultured in HG, HG+A-769662, and HG+Dorsomorphin for 72 h. (d) Fasting blood glucose was recorded every week beginning at 8 weeks of age. The data are expressed as the mean ± SD (*n* = 5/group). ^∗∗^*p* < 0.01, *vs*. the db/m group at 8 weeks; ^##^*p* < 0.01, *vs*. the db/db group at 8 weeks; ^&&^*p* < 0.01, *vs*. the db/m group at 16 weeks; ^%%^*p* < 0.01, *vs*. the db/db group at 16 weeks; ^∧∧^*p* < 0.01, *vs*. the db/m group at 24 weeks; ^++^*p* < 0.01, *vs*. the db/db group at 24 weeks. (e) Serum creatinine and (f) urine ACR were detected at different weeks. The data are expressed as the mean ± SD (*n* = 5/group). ^∗∗^*p* < 0.01, *vs*. the db/m group at 16 weeks; ^#^*p* < 0.05, *vs*. the db/db group at 16 weeks; ^&&^*p* < 0.01, *vs*. the db/m group at 24 weeks; ^%%^*p* < 0.01, *vs*. the db/db group at 24 weeks. (g) HE staining, (h) PAS staining, and (i) Masson trichrome staining were performed in renal cortex sections from the db/m group, db/db group, and db/db+Met group at different weeks. Scale bars = 50 *μ*m.

As is widely known, metformin could change the biological functions by regulating the expression of AMPK in many types of cells. Thus, we further explored whether AMPK participated in the regulation of MBNL or miR-130a by metformin. Firstly, we investigated the effect of metformin on AMPK, and the expressions of AMPK in HK-2 cells were detected by Western blot. Results showed that compared with the HG group, the AMPK and p-AMPK protein expressions were significantly increased in the HG+Met group (Figure 6(a)). We then studied the effects of activated or inhibited AMPK on the MBNL1/miR-130a-3p pathway in HK-2 cells. The expressions of MBNL1 were detected by Western blot, and the expressions of miR-130a-3p were detected by qRT-PCR. However, the expression of miR-130a-3p and MBNL1 showed no statistical differences between the HG group and the AMPK activator treatment (HG+A-769662) group or the AMPK inhibitor treatment (HG+ Dorsomorphin) group (Figures 6(b) and 6(c)). This result indicated that there may be no obvious interactions between AMPK and MBNL1 or miR-130a-3p in renal tubular epithelial cells.

### 3.6. STAT3 Is a Target Gene of miR-130a-3p. Metformin Enhanced the Negative Regulation of STAT3 by Upregulating miR-130a-3p and Thus Reduced the High-Glucose-Induced Senescence of Renal Tubular Epithelial Cells

A potential binding site on miR-130a-3p for STAT3 was predicted by targetScan and miRanda software, which was then confirmed by dual-luciferase reporter gene assay. These investigations were performed under HG conditions. Results showed that luciferase activity was significantly decreased in the STAT3-Wt+miR-130a-3p group compared with the STAT3-Wt+miR-130a-3p-NC group, but there were no statistical differences between the STAT3-Wt+miR-130a-3p-NC group and STAT3-Mut+miR-130a-3p-NC group or the STAT3-Mut+miR-130a-3p group (Figure 5(f)). The effects on the senescence of renal tubular epithelial cells were detected by cotransfection of miR-130a-3p and STAT3. The P21 levels were significantly increased in the miR-130a-3p(+)+STAT3(+) group compared with the miR-130a-3p(+)+STAT3(+)-NC group. This indicated that overexpressed STAT3 could reverse the miR-130a-3p(+)-induced inhibition on the senescence of renal tubular epithelial cells. Compared with the miR-130a-3p(+)+STAT3(+) group, protein expression of P21 was decreased in the Met+miR-130a-3p(+)+STAT3(+) group (Figure 5(g)).

### 3.7. Metformin Reduced the Senescence of Renal Tubular Epithelial Cells in db/db Mice with Diabetic Nephropathy via the MBNL1/miR-130a-3p/STAT3 Pathway

In this study, we observed the renal changes of mice in the db/m group (control group) and db/db group and db/db mice that were treated with the metformin (db/db+Met) group for 24 weeks. Renal changes were detected at 8, 16, and 24 weeks. The fasting blood glucose was significantly higher in the db/db group than in the db/m group. The db/db+Met group showed lower blood glucose than the db/db group (Figure 6(d)). At 8 weeks, there were no statistical differences in Serum creatinine and urine albumin-to-creatinine ratio (ACR) among three groups. At 16 weeks and 24 weeks, serum creatinine and urine ACR were significantly increased in the db/db group than in the db/m group and significantly decreased in the db/db+Met group than in the db/db group (Figures 6(e) and 6(f)). At 8 weeks, there were no obvious pathological changes in the three groups. At 16 weeks and 24 weeks, compared with the db/m group, renal hematoxylin eosin (HE) staining showed glomerular hypertrophy, Periodic Acid Schiff (PAS) staining indicated increased glomerular mesangial matrix, and Masson trichrome staining revealed aggravated interstitial fibrosis of renal tubules in the db/db group; the abovementioned pathological changes were alleviated by metformin comparing db/db+Met group with the db/db group (Figures 6(g)–6(i)).

**Figure 7:**
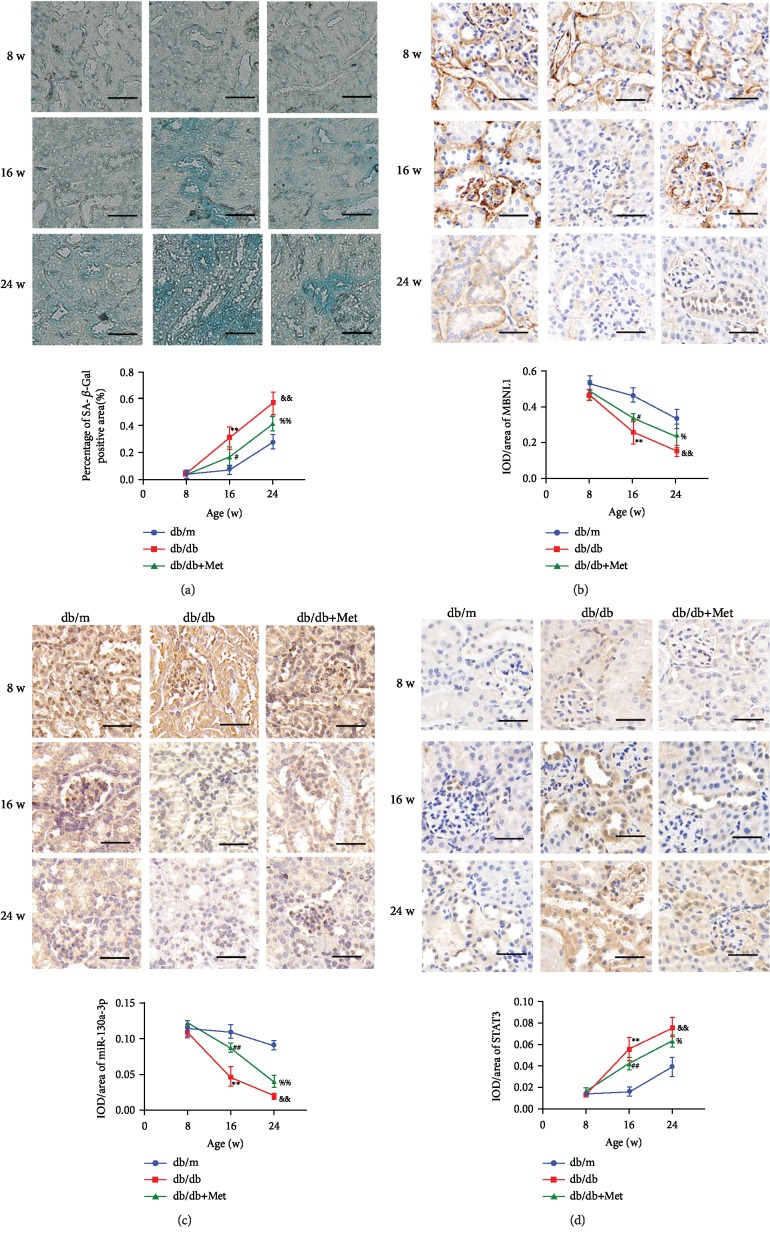
Metformin reduced the senescence of renal tubular epithelial cells in db/db mice through MBNL1/miR-130a-3p/STAT3 signaling. (a) SA-*β*-gal staining, (b) MBNL1 immunostaining, (c) *in situ* hybridization for miR-130a-3p, and (d) STAT3 immunostaining were performed in renal cortex sections from the db/m group, db/db group, and db/db + Met group at different weeks. The data are expressed as the mean ± SD (*n* = 5/group). ^∗∗^*p* < 0.01, *vs*. the db/m group at 16 weeks; ^#^*p* < 0.05, *vs*. the db/db group at 16 weeks; ^##^*p* < 0.01, *vs*. the db/db group at 16 weeks; ^&&^*p* < 0.01, *vs*. the db/m group at 24 weeks; ^%^*p* < 0.05, *vs*. the db/db group at 24 weeks; ^%%^*p* < 0.01, *vs*. the db/db group at 24 weeks. Scale bars = 50 *μ*m.

At 8 weeks, there were no statistical differences in the *β*-galactosidase-positive staining rate of renal tubular epithelial cells among the three groups. At 16 weeks and 24 weeks, the *β*-galactosidase-positive staining rate was significantly increased in the db/db group than in the db/m group and significantly decreased in the db/db+Met group than in the db/db group (Figure 7(a)). At 8 weeks, there were no statistical differences in the expression of MBNL1, miR-130a-3p, and STAT3 in the cells among the three groups. At 16 weeks and 24 weeks, compared with the db/m group, the expression of MBNL1 and miR-130a-3p were significantly decreased, and STAT3 expression was markedly increased in the db/db group; metformin could alleviate the abovementioned phenomenon as compared to the db/db + Met group with the db/db group(Figures 7(b)–7(d)).

## 4. Discussion

**Figure 8:**
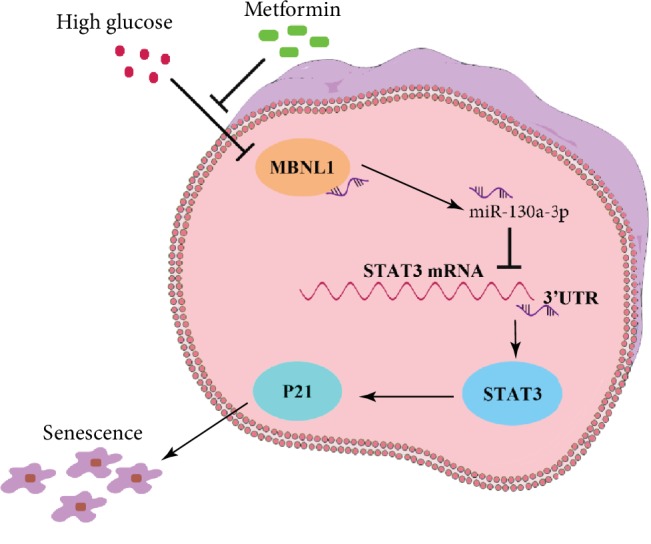
The schematic summary of the regulatory mechanisms. Metformin reduces the senescence of renal tubular epithelial cells in diabetic nephropathy via the MBNL1/miR-130a-3p/STAT3 pathway.

This study has demonstrated that metformin can reduce the high-glucose-induced senescence of renal tubular epithelial cells and thus suppress high-glucose-induced renal lesions. In the process of senescence of renal tubular epithelial cells caused by high glucose, leading to renal tubular lesions, MBNL1 and miR-130a-3p were weakly expressed, and STAT3 was highly expressed. As MBNL1 can specifically bind to miR-130a-3p, thus increasing its stability, low expression of MBNL1 leads to a decrease in the stability of miR-130a-3p; furthermore, miR-130a-3p bound to the 3′UTR of STAT3 and negatively regulated its expression, thus reducing the senescence of renal tubular epithelial cells. Metformin enhanced the stability of miR-130a-3p by upregulating MBNL1 expression, as well as upregulating miR-130a-3p expression, thus strengthening the negative regulation on STAT3, inhibiting STAT3 expression, and reducing renal tubular lesions caused by the high-glucose-induced senescence of renal tubular epithelial cells (Figure 8).

The relevant underlying mechanism of diabetic renal lesions has been a long-standing concern of investigators. Cell senescence has been recently shown to play a role in diabetic renal lesions. High glucose can induce the enhanced activity of *β*-galactosidase and increase the protein expression of P21 in glomerular mesangial cells, thus promoting cell senescence [31, 32]. Our study demonstrated that high glucose can contribute to the enhanced activity of *β*-galactosidase and the increased protein expression of P21 in renal tubular epithelial cells (promoting cell senescence), while metformin can effectively inhibit these high-glucose-induced effects, and therefore reduce senescence. Our results are similar to other reports; for example, high glucose can promote the senescence of retinal pigment epithelial cells and mesenchymal stem cells [33, 34]. Metformin inhibits the high-glucose-induced senescence of vascular endothelial cells by upregulating SIRT1 expression and thus improving vascular lesions [35]; metformin also suppresses the high-glucose-induced senescence of fibroblasts by downregulating NF-*κ*B expression [36].

The role of RBPs in diabetes has attracted increasing interest from investigators. Ybx1 was shown to be weakly expressed in the kidneys of diabetic mice, suggesting a role for this RBP in diabetes [37]. According to the study by Guo et al., QKI5 was weakly expressed in the heart of diabetic mice, and overexpression of QKI5 could prevent cardiac ischemic injury in diabetes [38]. Our study revealed that MBNL1 was weakly expressed in the high-glucose condition and metformin can reduce the high-glucose-induced senescence by upregulating MBNL1 expression.

We found that miR-130a-3p was also weakly expressed in high-glucose condition. Metformin could reduce senescence by upregulating miR-130a-3p. Previous studies have shown that miR-130a-3p could alleviate high-glucose-induced podocyte dysfunction in mice [39]. Another study suggested that miR-130a-3p was weakly expressed in the livers of diabetic mice, and overexpression of miR-130a-3p could inhibit hepatic fatty degeneration of diabetic mice [40]. The abovementioned reports have revealed that low miR-130a-3p expression is associated with multiple organ lesions in diabetes. Metformin can play a role in preventing the senescence by upregulating miR-130a-3p and thus inhibit high-glucose-induced renal tubular lesions.

Studies have shown that RBPs can interact with miRNAs to play several biological functions. RBPs function by binding to miRNAs to alter their stability; for example, ILF3 binds to miR-144 to increase its stability [41]. Moreover, FXR1 regulates the biological behavior of glioma cells by increasing thestability of MIR17HG [42]. In this study, RBPDB software, RIP and pull-down assays showed that MBNL1 could bind to miR-130a-3p and overexpressed MBNL1 did not change the nascent RNA levels but prolonged the half-life of miR-130a-3p, indicating that overexpressed MBNL1 could increase the stability of miR-130a-3p. This study also showed that silencing miR-130a could overcome the effects of MBNL1, while upregulating miR-130a could further promote the effects of MBNL1. These findings fully support the notion that MBNL1 specifically binds to miR-130a-3p to increase its stability. Furthermore, this study indicate that metformin increases the stability of miR-130a-3p by upregulating MBNL1, which in turn upregulates miR-130a-3p expression, thus playing an inhibitory role under high glucose condition.

miRNAs can bind to the mRNA 3′UTR of target genes and negatively regulate their expression. For example, miR-298 binds to the STAT3 mRNA 3′UTR to regulate the growth of liver cancer cells [43]. Some studies have suggested that high glucose can increase STAT3 expression in human renal tubular epithelial cells and human peritoneal mesothelial cells [44, 45], and metformin has been shown to inhibit the development of pancreatic cancer and esophageal squamous cell carcinoma by downregulating STAT3 expression [46]. In our study, on the basis of predicting a potential binding site of miR-130a-3p and STAT3 3′UTR with targetScan and miRanda software, the targeted binding between miR-130a-3p and STAT3 mRNA 3′UTR was validated by the dual-luciferase reporter gene assay. Moreover, STAT3 was highly expressed in the high-glucose condition. At the same time, miR-130a-3p could decrease STAT3 expression and inhibit senescence. Furthermore, STAT3 could reverse the effects of miR-130a-3p. At last, metformin increased miR-130a-3p expression but decreased STAT3 expression in renal tubular epithelial cells. Our results indicated that metformin could reduce senescence by upregulating miR-130a-3p and downregulating STAT3.

Several studies have shown that db/db mice are a classic animal model for studying diabetic nephropathy [47, 48]. Here, we dynamically observed the changes in the db/m, db/db, and db/db+Met groups at 8, 16, and 24 weeks. At 8 weeks, blood glucose was significantly increased, but there were no obvious pathological changes; the *β*-galactosidase positive staining rate and the expression of MBNL1, miR-130a-3p, and STAT3 were unchanged in the db/db and db/db+Met groups, compared with the db/m group. An increase in blood glucose in db/db mice at 8 weeks was also reported by Liu et al. [49]. At 16 and 24 weeks, compared with the db/m group, levels of blood glucose, serum creatinine, urine ACR, and the *β*-galactosidase positive staining rate were gradually increased, and renal pathological changes were progressively aggravated in the db/db group, showing a consistent trend between the senescence of renal tubular epithelial cells and the pathological changes. Meanwhile, there was a decrease in the expression of MBNL1 and miR-130a-3p, but a gradual increase in STAT3 expression in renal tubular epithelial cells of the db/db group. Meanwhile, whenmetformin was administered, those changes were alleviated as compared with the db/db group. Our results suggest that the metformin/MBNL1/miR-130a-3p/STAT3 pathway is involved in regulating the senescence and pathological changes in renal tubular epithelial cells in diabetes. Similar to our study results, one study reported that metformin can alleviate renal fibrosis in diabetic nephropathy mice by changing miR-192 expression [50]. Our study results indicate that MBNL1, miR-130a-3p, and STAT3 may become new targets for treating diabetic renal tubular lesions.

In conclusion, through investigations on renal tubular epithelial cells and db/db mice, this study has demonstrated an important regulatory role of the metformin/MBNL1/miR-130a-3p/STAT3 pathway in the process of high-glucose-induced senescence of renal tubular epithelial cells. Our results add a new theoretical and experimental basis for the pathogenesis of diabetic nephropathy and provide new ideas for therapy of this disease.

## Data Availability

The data used to support the findings of this study are available from the corresponding authors upon request.

## Abbreviations

ESRD: End-stage renal disease
RBPs: RNA-binding proteins
STAT3: Signal transmission and transcription activation factor 3
NG: Normal glucose
HG: High glucose
MA: Mannitol
DMEM/F-12: Dulbecco's Modified Eagle Medium/Nutrient Mixture F-12
sh: Short hairpin
NC: Negative control
PVDF: Polyvinylidene difluoride
SDS-PAGE: Sodium dodecyl sulfate-polyacrylamide gel electrophoresis
EU: Ethynyl uridine
Met: Metformin
RIP: RNA-binding protein immunoprecipitation
ACR: Albumin-to-creatinine ratio.

## Additional Points

*Summary Statement*. Metformin reduces senescence via MBNL1/miR-130a-3p/STAT3 pathway in DN.

## Supplementary Materials

Supplementary Materials “Western blot”. Uncropped, unedited blot from all figures.
