# Correction to “Metformin Reduces the Senescence of Renal Tubular Epithelial Cells in Diabetic Nephropathy via the MBNL1/miR‐130a‐3p/STAT3 Pathway”

**DOI:** 10.1155/omcl/9781279

**Published:** 2026-04-01

X. Jiang, X. Ruan, Y. Xue, S. Yang, M. Shi, and L. Wang, “Metformin Reduces the Senescence of Renal Tubular Epithelial Cells in Diabetic Nephropathy via the MBNL1/miR‐130a‐3p/STAT3 Pathway,” *Oxidative Medicine and Cellular Longevity* 2020, no. 1 (2020): 8708236, https://doi.org/10.1155/2020/8708236.

In the article titled “Metformin Reduces the Senescence of Renal Tubular Epithelial Cells in Diabetic Nephropathy via the MBNL1/miR‐130a‐3p/STAT3 Pathway,” there were multiple errors. These errors are shown and corrected below:

Error in Figure **3**c:

Figure 3c mistakenly contained an additional GAPDH band. This error was made during figure preparation. The correct Figure 3 is as follows:

Figure 3miR‐130a‐3p was downregulated in senescent HK‐2 cells, and metformin reduced cell senescence in HK‐2 cells by upregulating miR‐130a‐3p. (a) qRT‐PCR was used to detect expression levels of miR‐130a‐3p in HK‐2 cells that were cultured in NG and HG for 72 h. The data are expressed as the mean ± SD (*n* = 3/group). 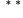 *p* < 0.01, vs. NG group. (b) The RNA expression levels of miR‐130a‐3p and STAT3 were detected in HK‐2 cells after miR‐130a‐3p overexpression or knockdown. (c) The protein expression levels of STAT3 and P21 were detected in HK‐2 cells. The data are expressed as the mean ± SD (*n* = 5/group). 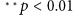, vs. the miR‐130a‐3p(+)‐NC group; ^#^ *p* < 0.05, vs. the miR‐130a‐3p(−)‐NC group; ^##^ *p* < 0.01, vs. the miR‐130a‐3p(−)‐NC group. (d) The RNA expression levels of miR‐130a‐3p and STAT3 were detected in HK‐2 cells after treatment with metformin. (e) The protein expression levels of STAT3 and P21 were detected in HK‐2 cells after treatment with metformin. The data are expressed as the mean ± SD (*n* = 3/group). 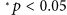, vs. the control group; 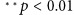, vs. the control group; ^#^ *p* < 0.05, vs. the Met group; ^##^ *p* < 0.01, vs. the Met group.

**Figure:**
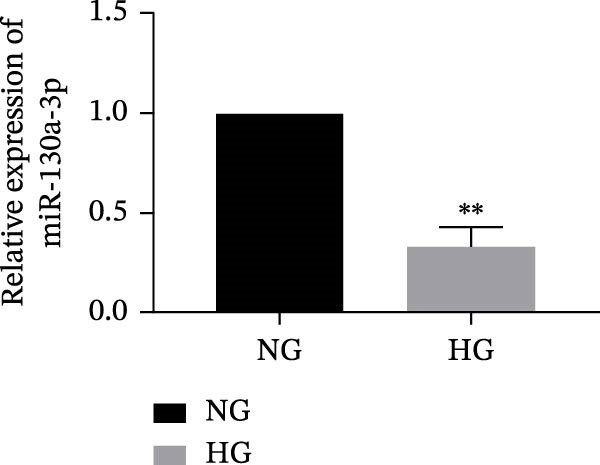
(a)

**Figure:**
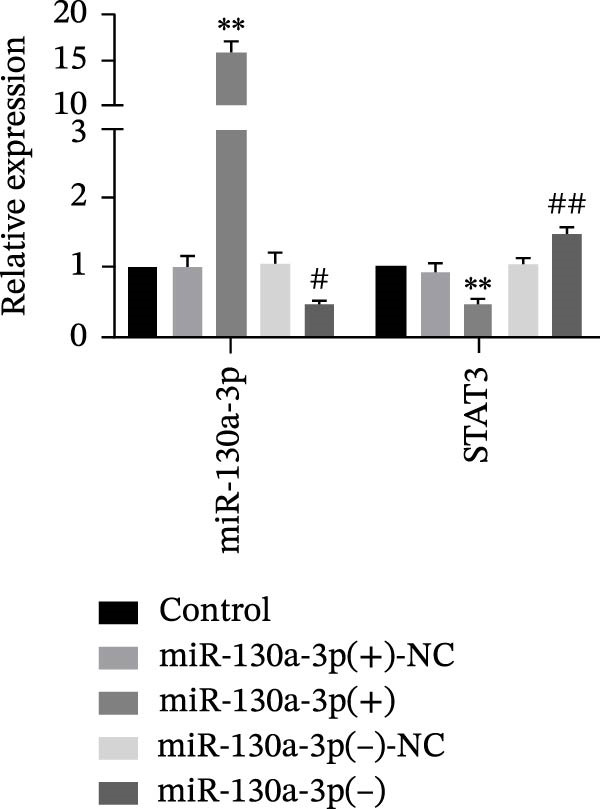
(b)

**Figure:**
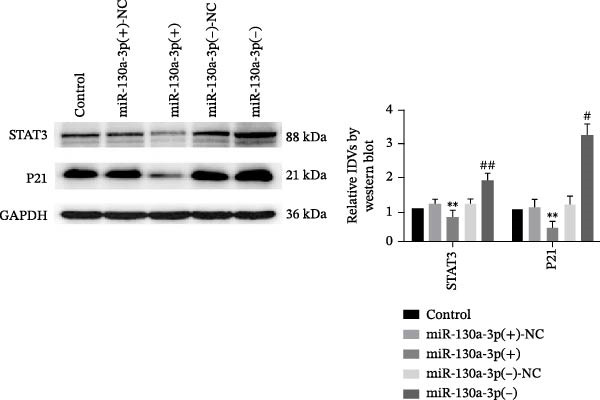
(c)

**Figure:**
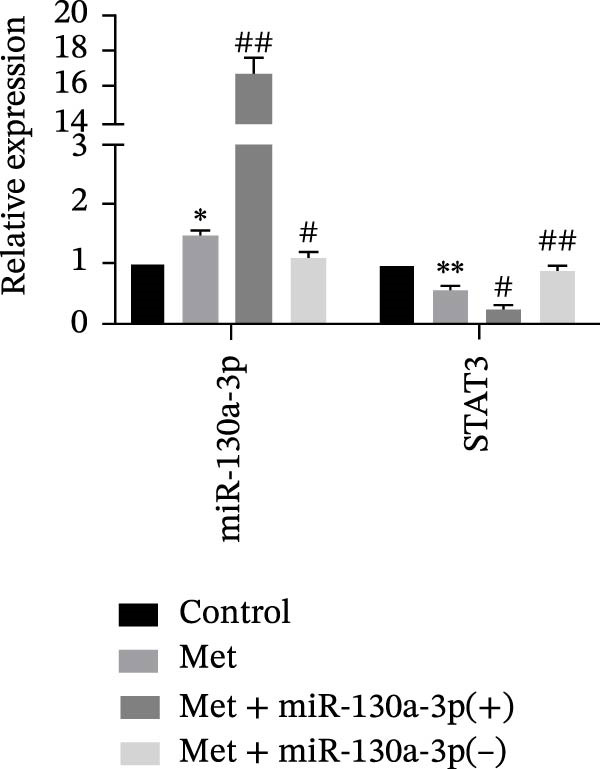
(d)

**Figure:**
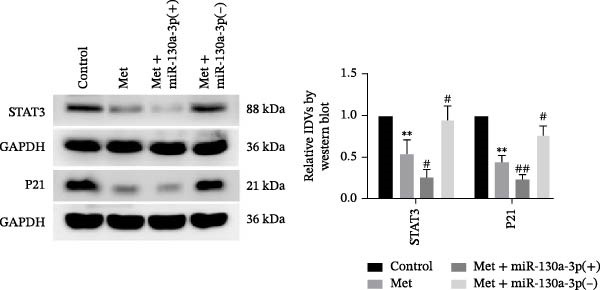
(e)

Error in Supporting Figure **3**C:

Supporting Figure 3C also contained an additional GAPDH band, which was introduced during figure assembly. The correct Supporting Figure 3C is as follows:

**Figure:**
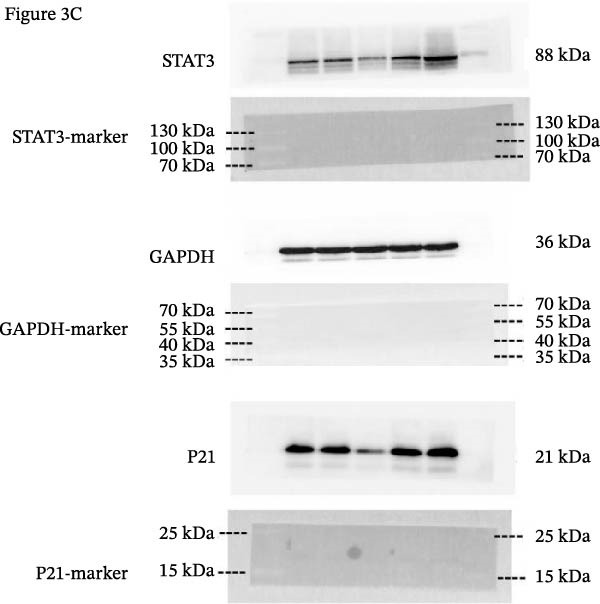


We apologize for these errors.

