# Supplementary material for: Metformin Reduces the Senescence of Renal Tubular Epithelial Cells in Diabetic Nephropathy via the MBNL1/miR-130a-3p/STAT3 Pathway
Source: Oxid Med Cell Longev. 2020 Feb 10;2020:8708236. doi: 10.1155/2020/8708236 (PMC7035567; doi:10.1155/2020/8708236)
Supplement: Supplementary Materials — “Western blot”. Uncropped, unedited blot from all figures. [file 8708236.f1.pdf]

Figure 1A

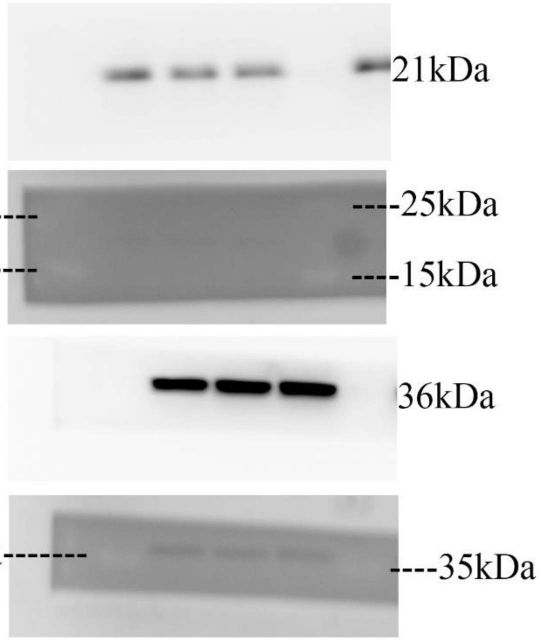

Figure 1B

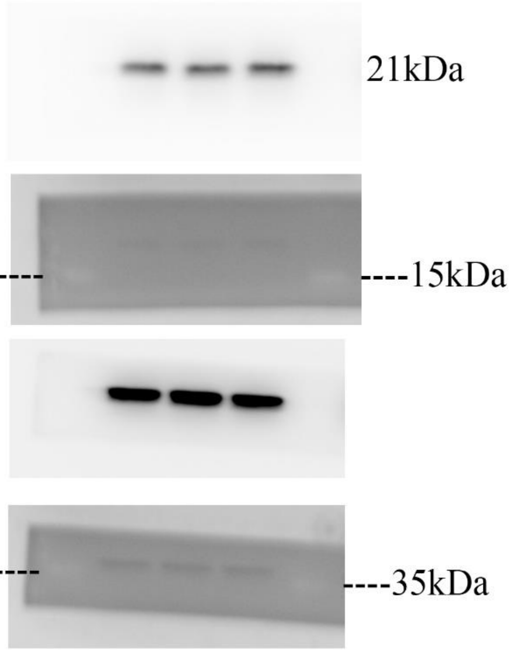

Figure 1C

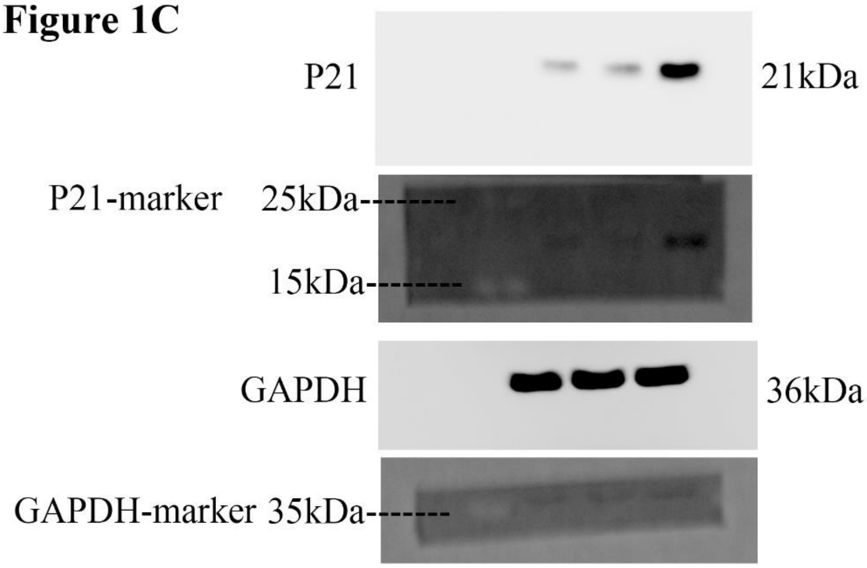

Figure 1E

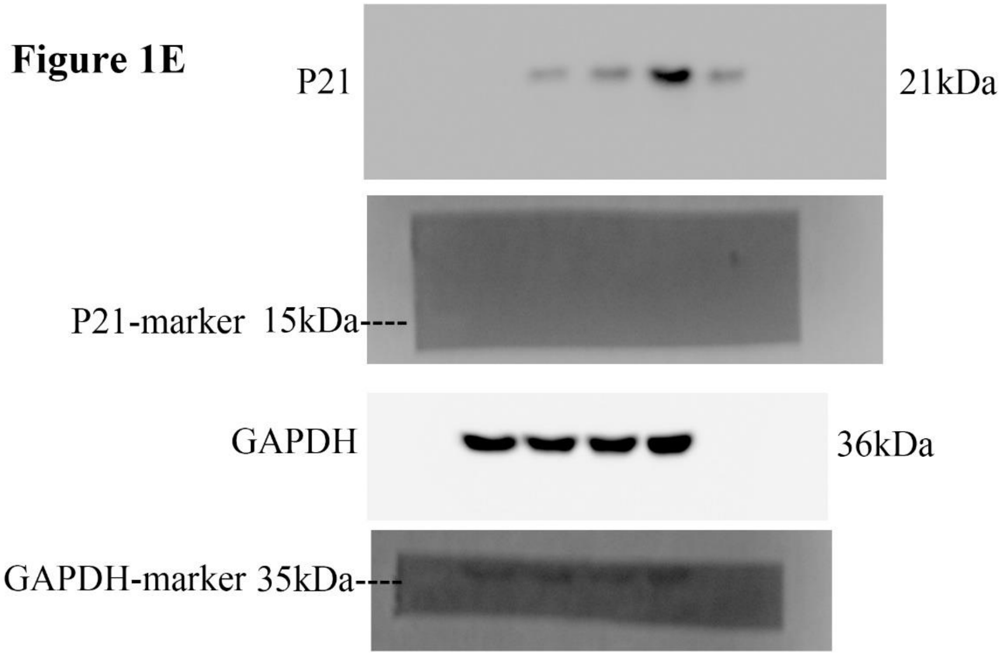

Figure 2B

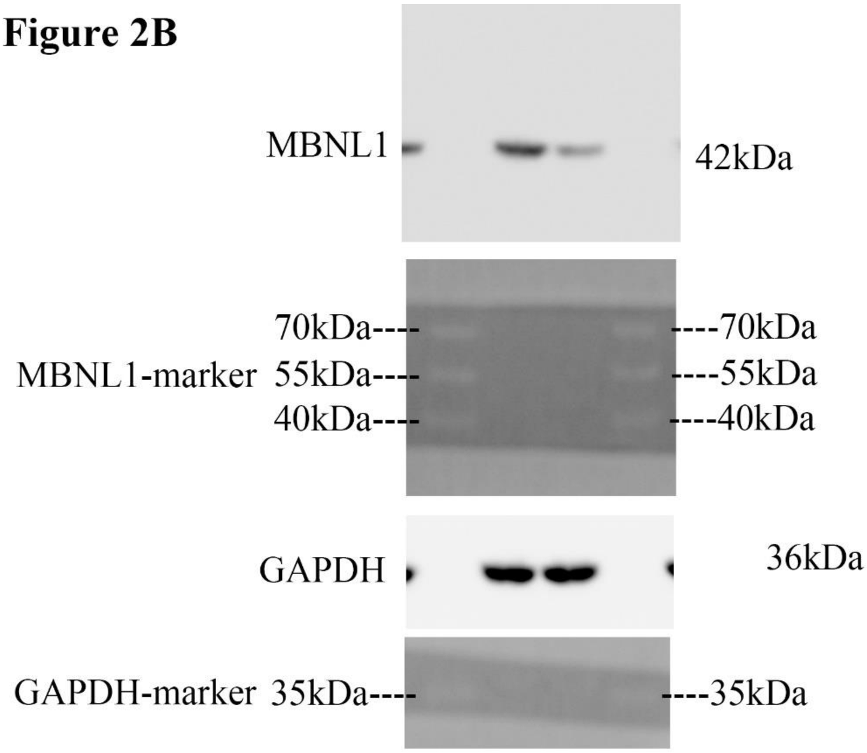

Figure 2D

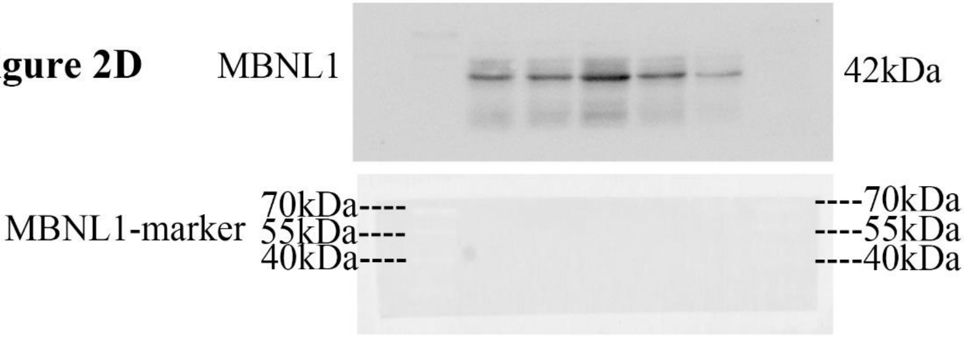

Figure 2D

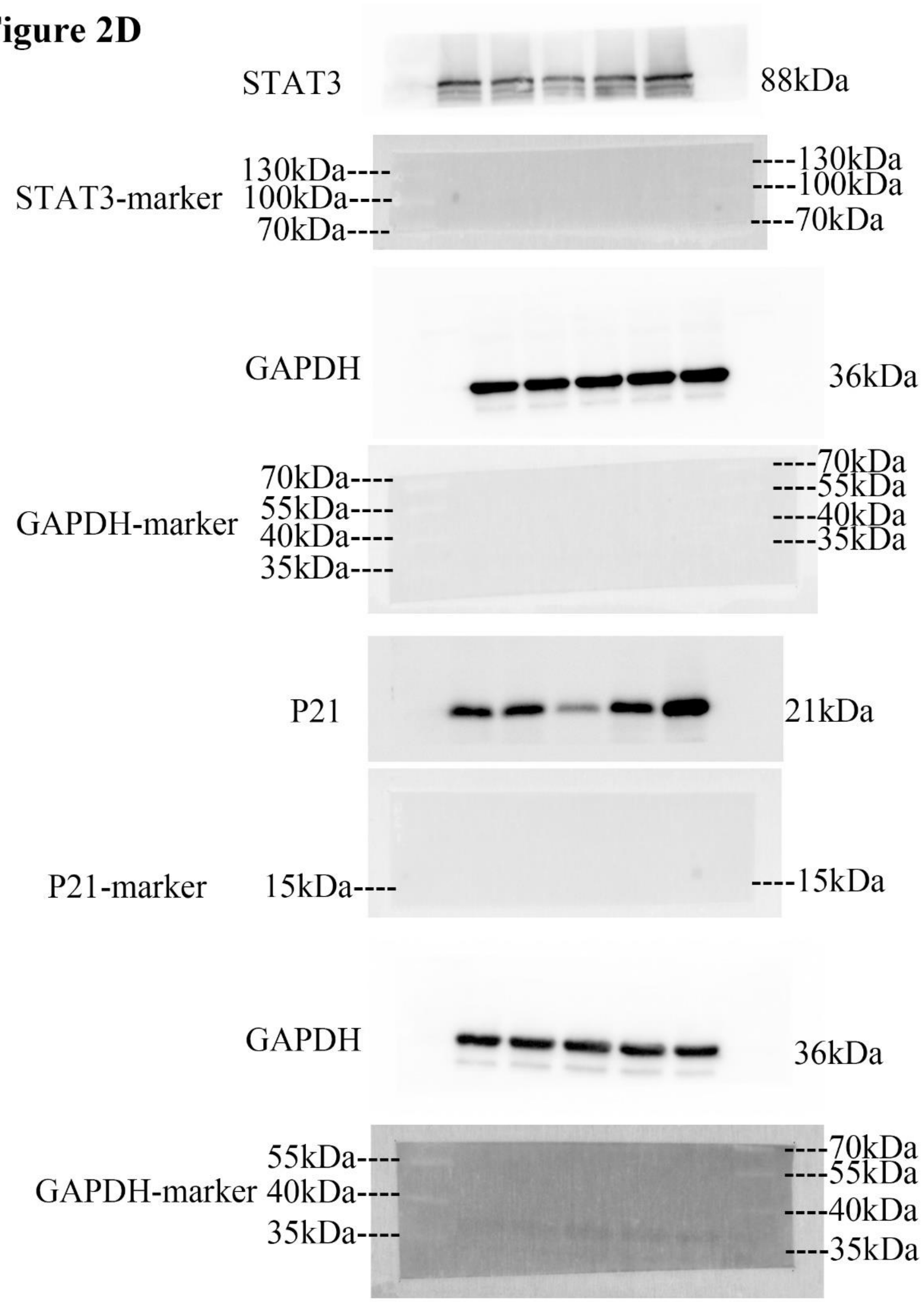
STAT3
88kDa
STAT3-marker
130kDa----
100kDa----
70kDa----
----130kDa
----100kDa
----70kDa
GAPDH
36kDa
GAPDH-marker
70kDa----
55kDa----
40kDa----
35kDa----
----70kDa
----55kDa
----40kDa
----35kDa
P21
21kDa
P21-marker
15kDa----
----15kDa
GAPDH
36kDa
GAPDH-marker
55kDa----
40kDa----
35kDa----
----70kDa
----55kDa
----40kDa
----35kDa

Figure 2F

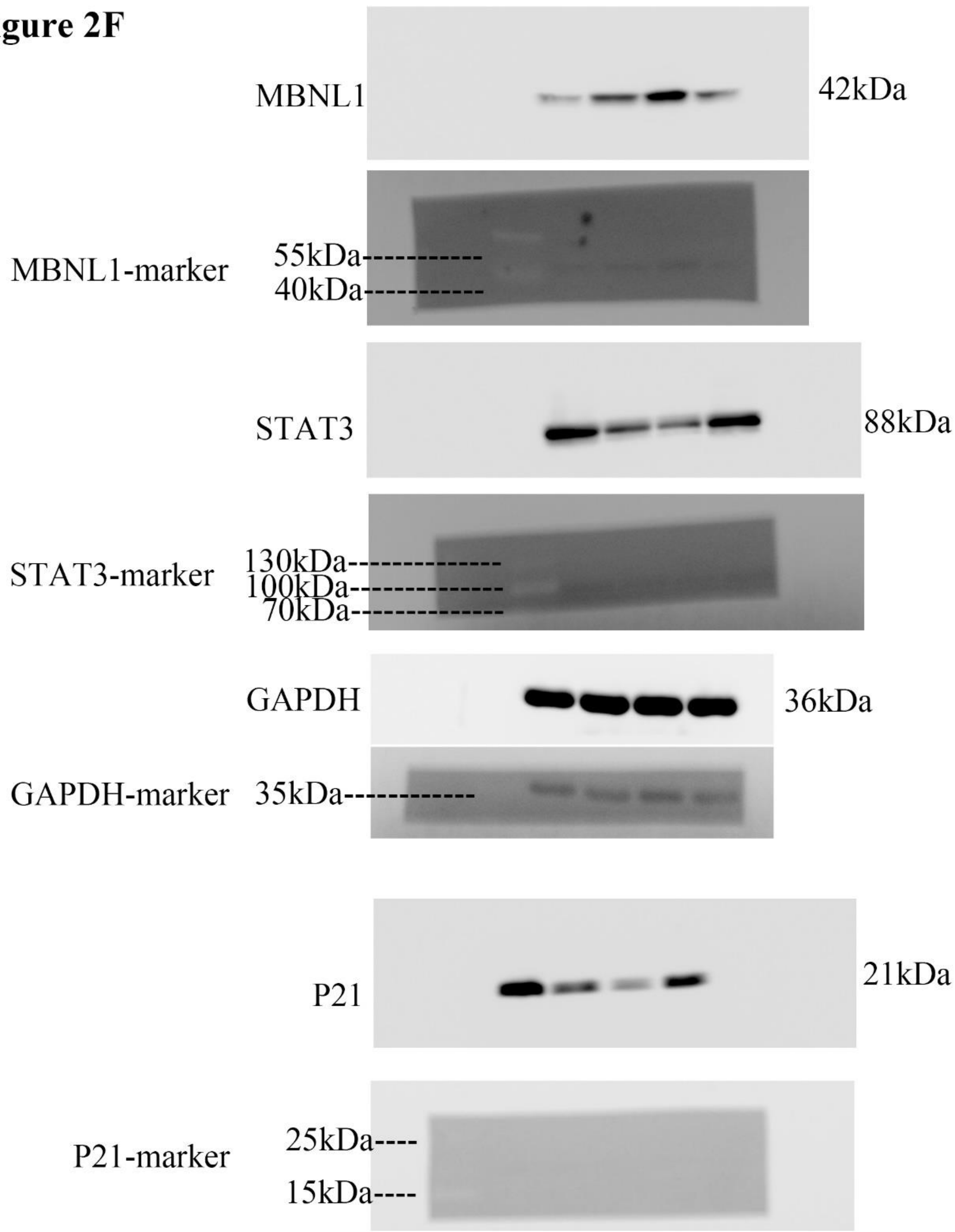
MBNL1
42kDa
MBNL1-marker
55kDa
40kDa
STAT3
88kDa
STAT3-marker
130kDa
100kDa
70kDa
GAPDH
36kDa
GAPDH-marker
35kDa
P21
21kDa
P21-marker
25kDa
15kDa

Figure 3C

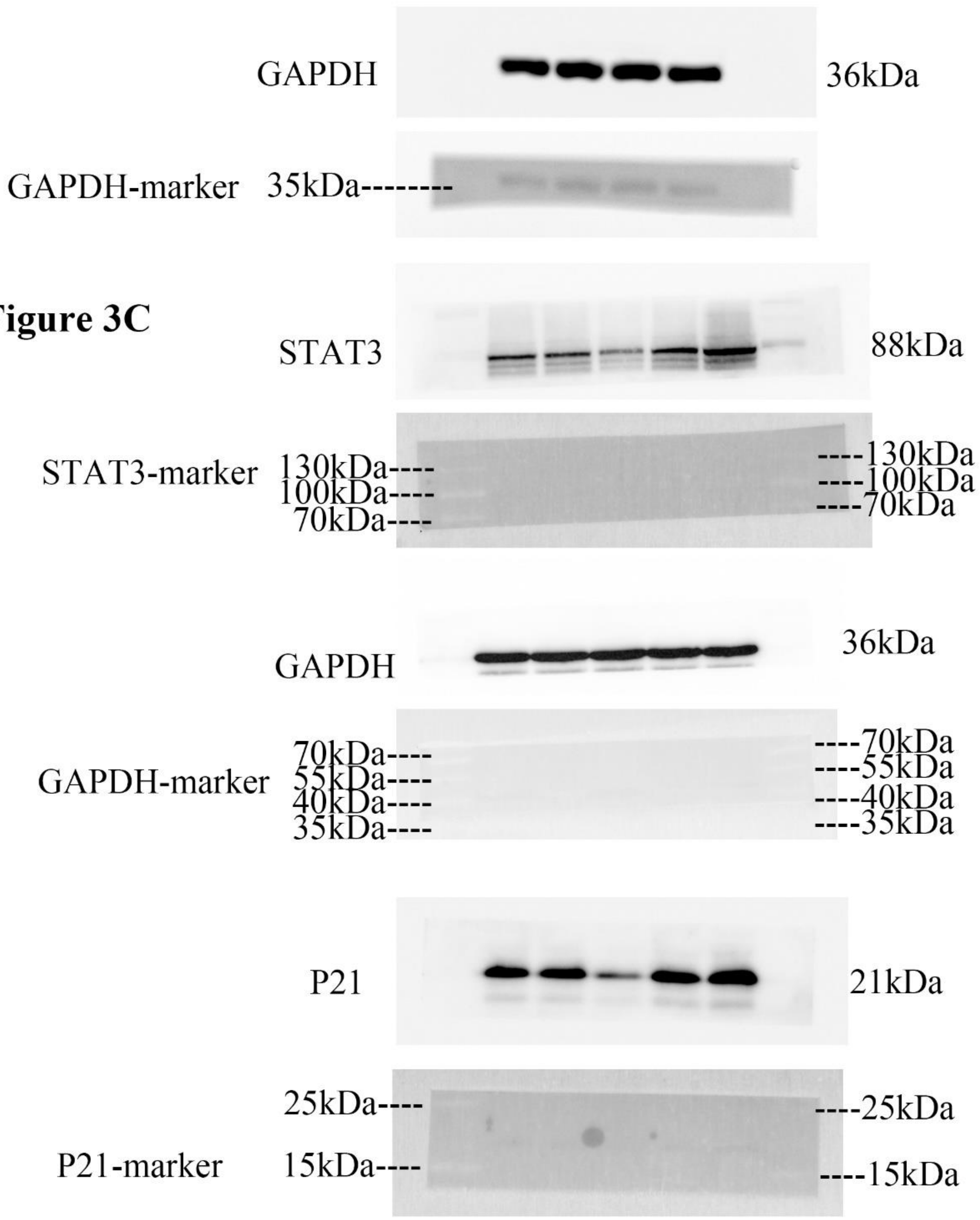
GAPDH
36kDa
GAPDH-marker
35kDa--------
STAT3
88kDa
STAT3-marker
130kDa----
100kDa----
70kDa----
----130kDa
----100kDa
----70kDa
GAPDH
36kDa
GAPDH-marker
70kDa----
55kDa----
40kDa----
35kDa----
----70kDa
----55kDa
----40kDa
----35kDa
P21
21kDa
P21-marker
25kDa----
15kDa----
----25kDa
----15kDa

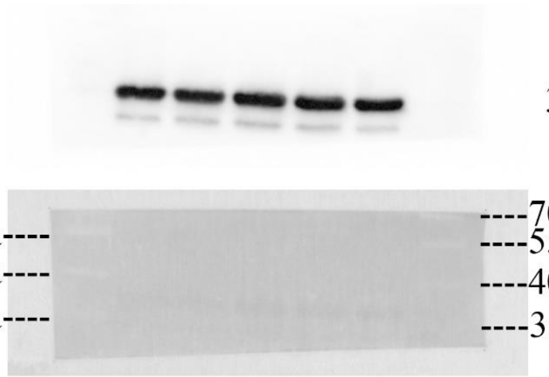
GAPDH
36kDa
GAPDH-marker
55kDa----
40kDa----
35kDa----
----70kDa
----55kDa
----40kDa
----35kDa

Figure 3E

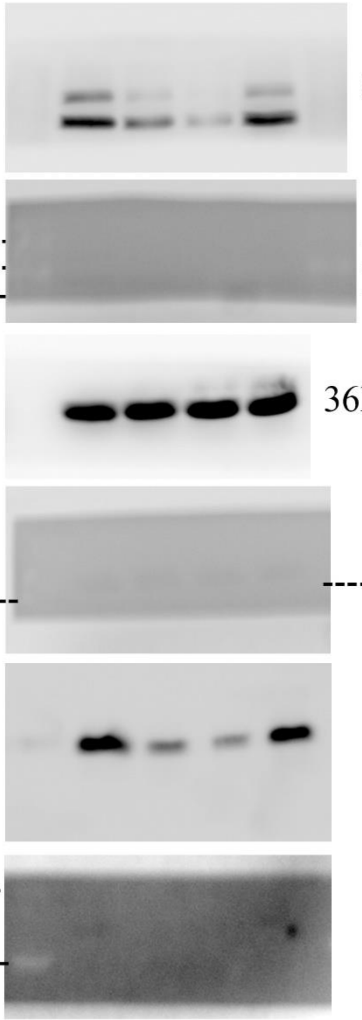
STAT3
88kDa
STAT3-marker
130kDa----
100kDa----
70kDa----
----130kDa
----100kDa
----70kDa
GAPDH
36kDa
GAPDH-marker
35kDa----
----35kDa
P21
21kDa
P21-marker
25kDa----
15kDa----

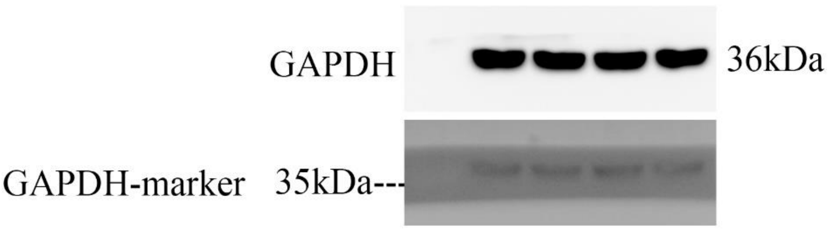

Figure 4B

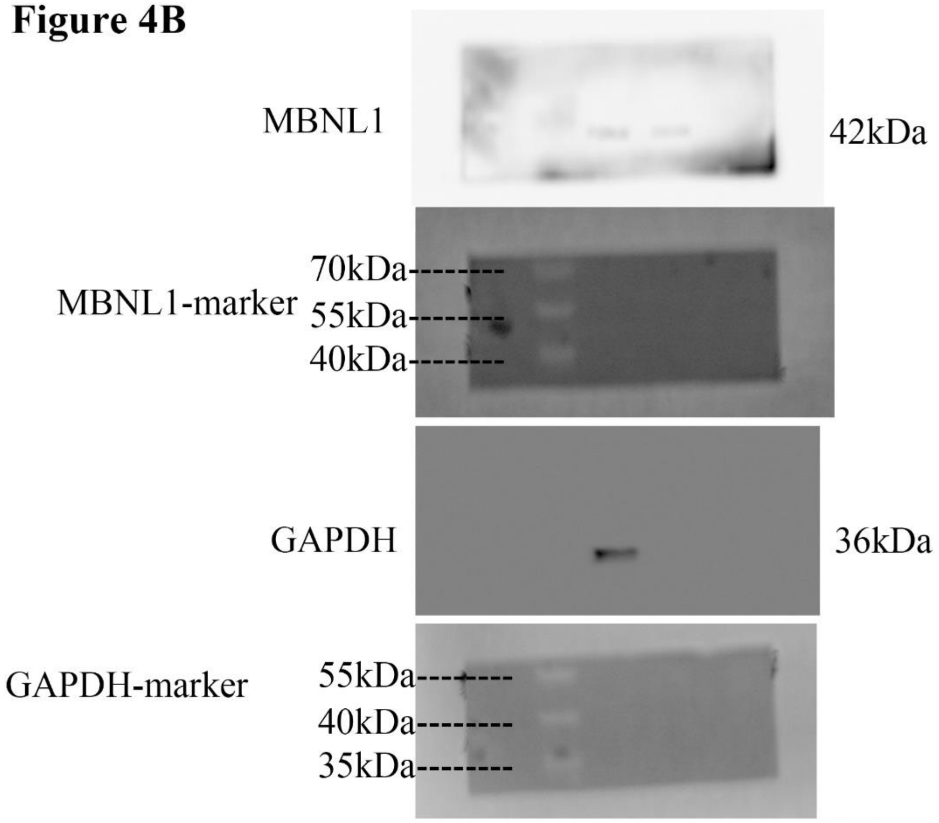

Figure 4E

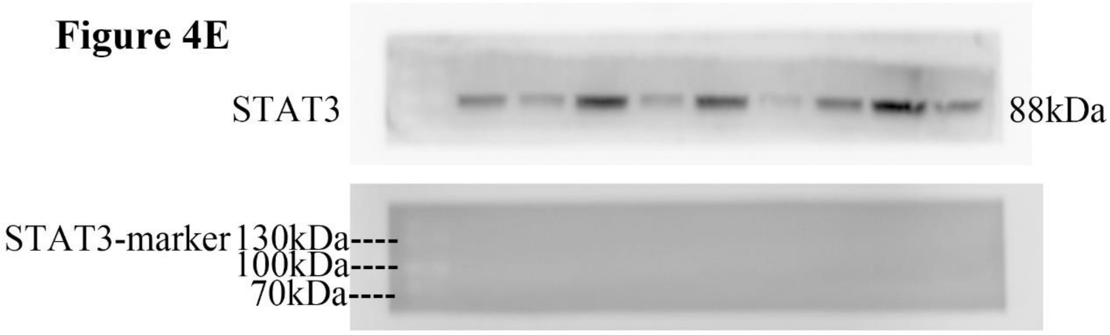

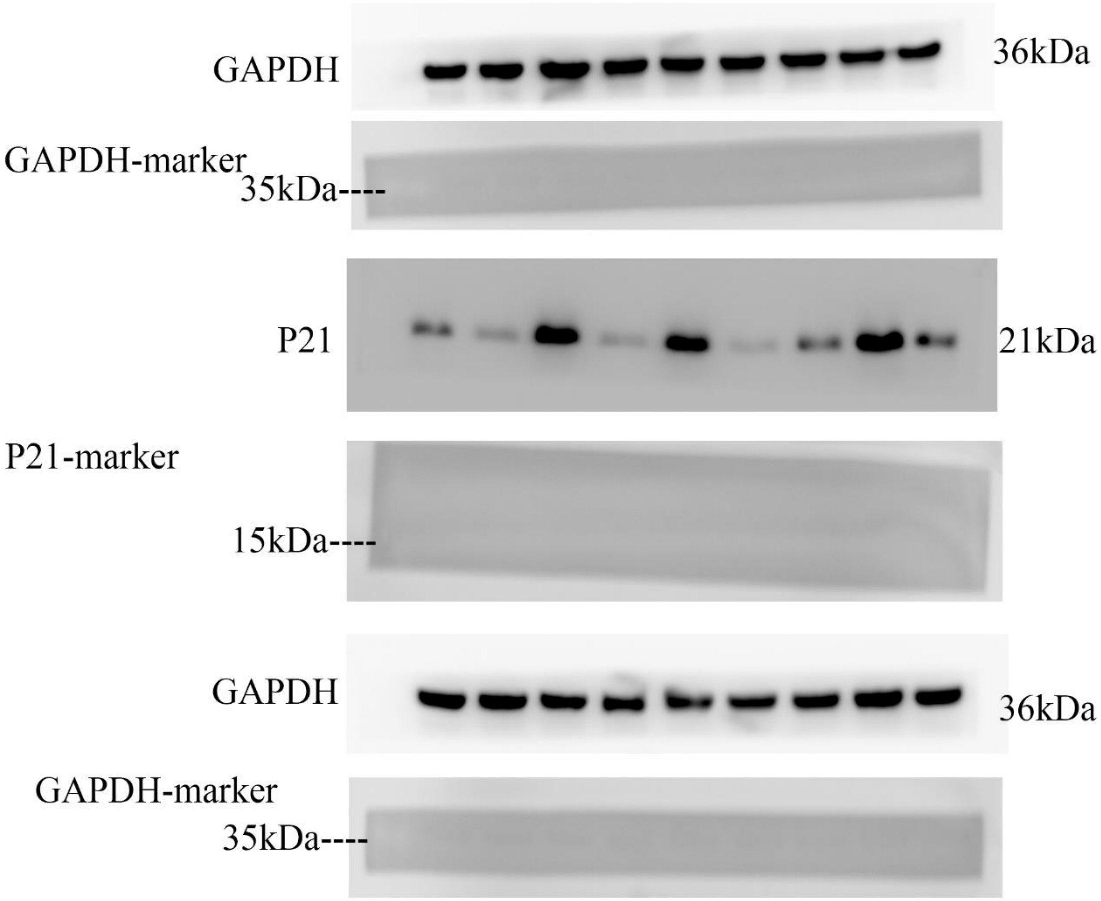
GAPDH
36kDa
GAPDH-marker
35kDa----
P21
21kDa
P21-marker
15kDa----
GAPDH
36kDa
GAPDH-marker
35kDa----

Figure 4F

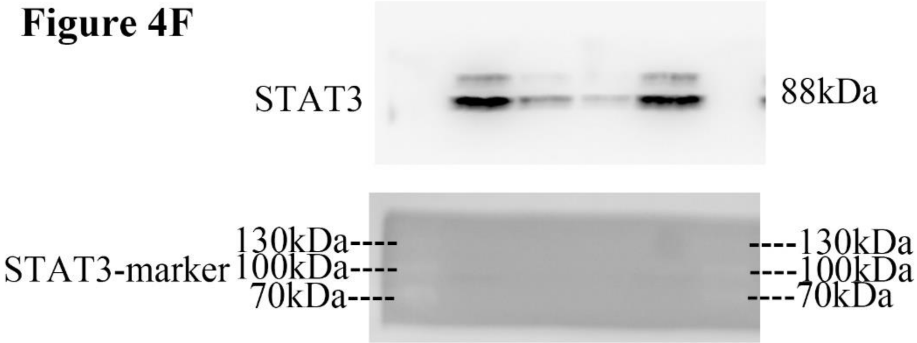
STAT3
88kDa
STAT3-marker
130kDa----
100kDa----
70kDa----
----130kDa
----100kDa
----70kDa

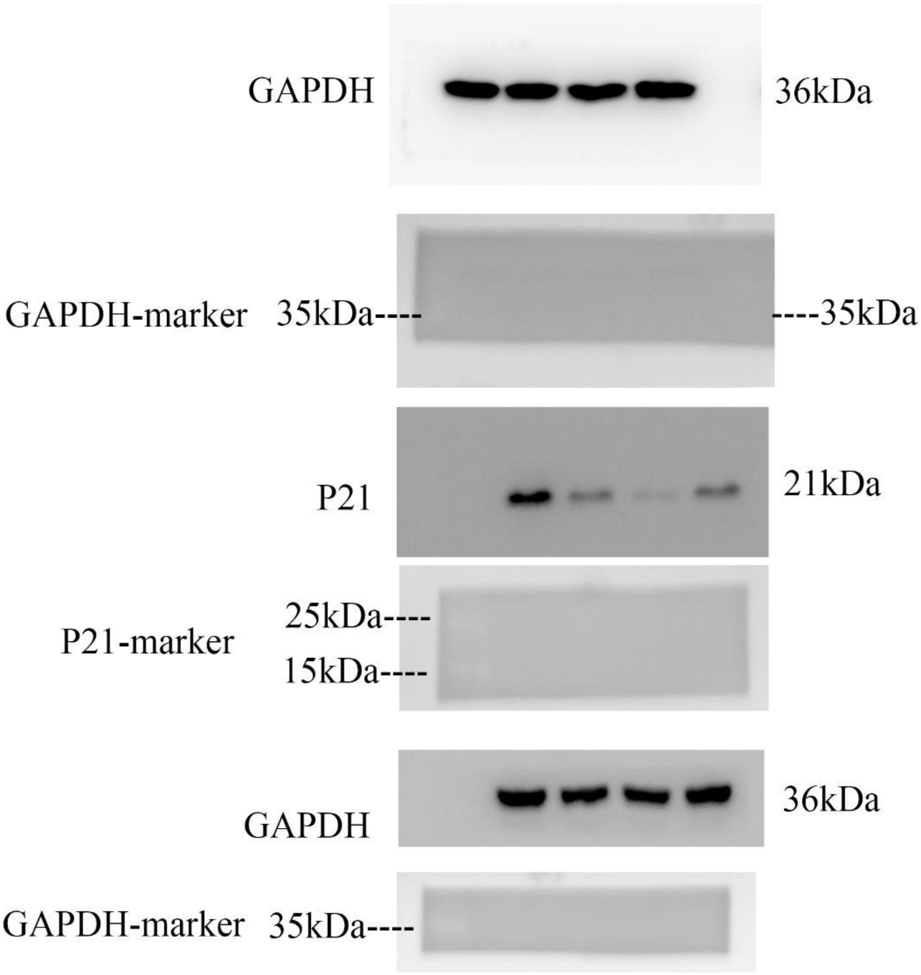
GAPDH
36kDa
GAPDH-marker
35kDa----
----35kDa
P21
21kDa
P21-marker
25kDa----
15kDa----
GAPDH
36kDa
GAPDH-marker
35kDa----

Figure 5B

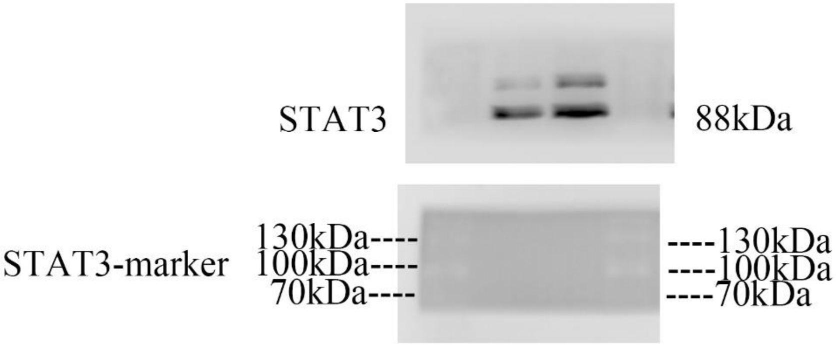
STAT3
88kDa
STAT3-marker
130kDa----
100kDa----
70kDa----
----130kDa
----100kDa
----70kDa

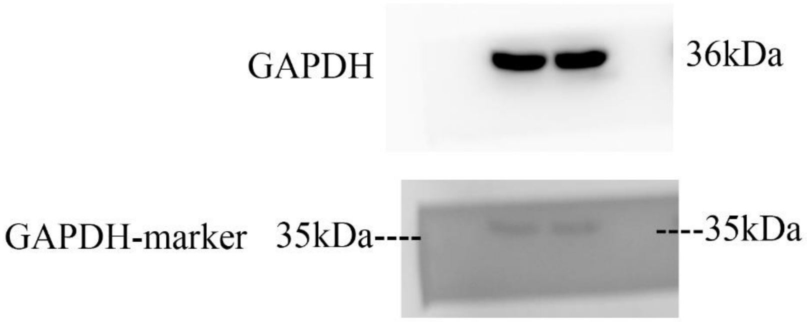
GAPDH
36kDa
GAPDH-marker
35kDa----
----35kDa

Figure 5D

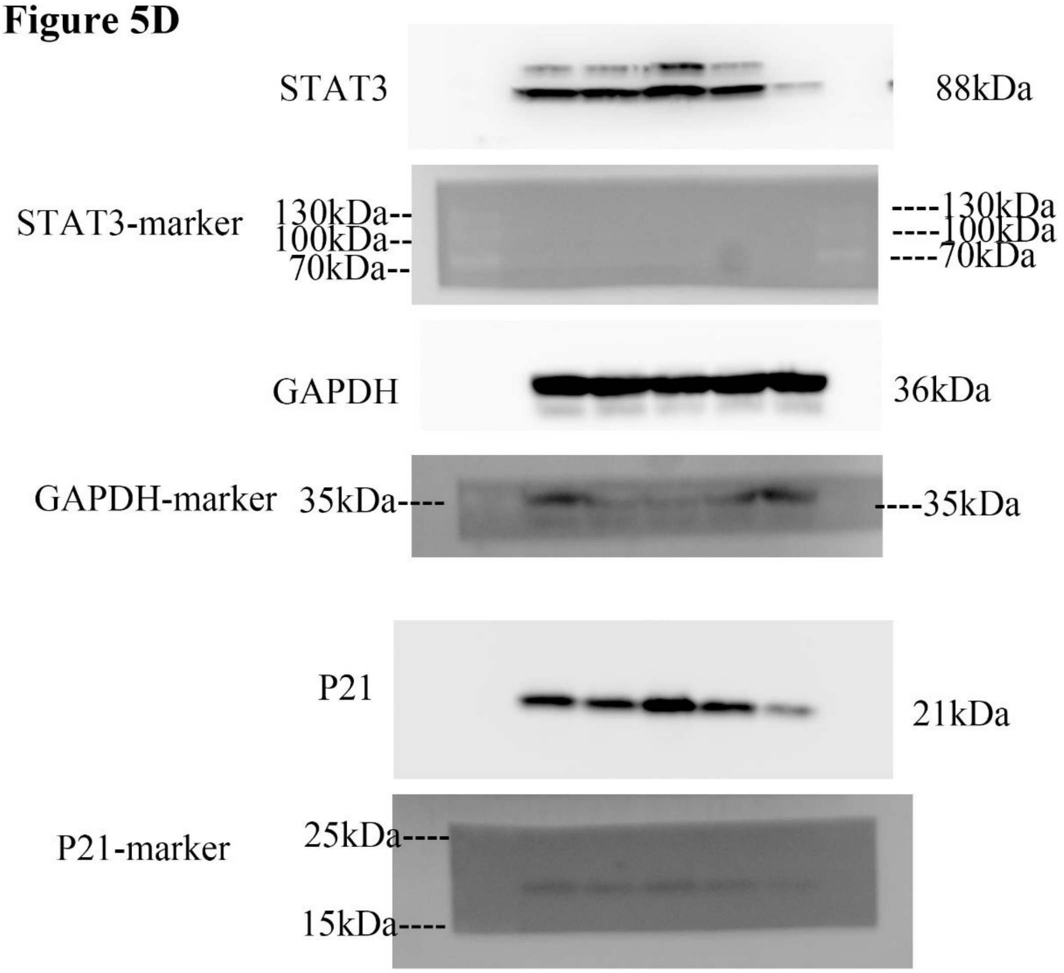
STAT3
88kDa
STAT3-marker
130kDa--
100kDa--
70kDa--
----130kDa
----100kDa
----70kDa
GAPDH
36kDa
GAPDH-marker
35kDa----
----35kDa
P21
21kDa
P21-marker
25kDa----
15kDa----

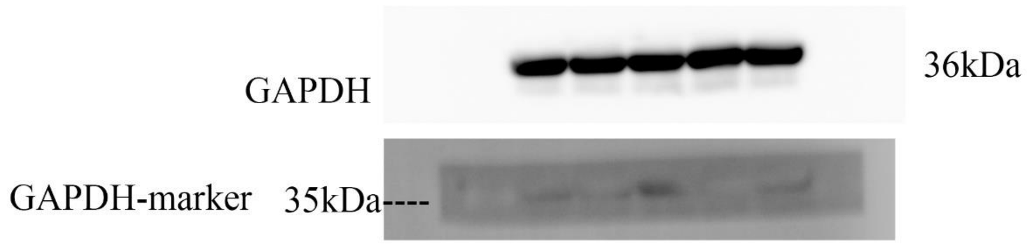
GAPDH
36kDa
GAPDH-marker
35kDa----

Figure 5E

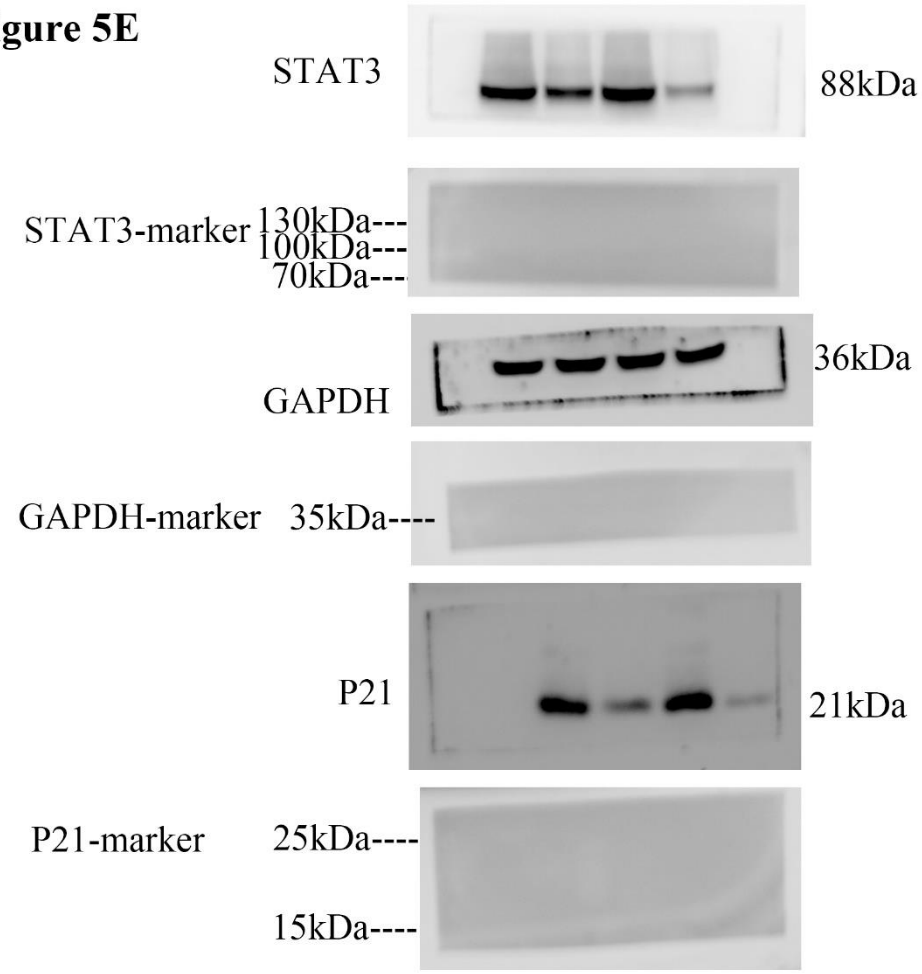
STAT3
88kDa
STAT3-marker
130kDa---
100kDa---
70kDa---
GAPDH
36kDa
GAPDH-marker
35kDa----
P21
21kDa
P21-marker
25kDa----
15kDa----

GAPDH 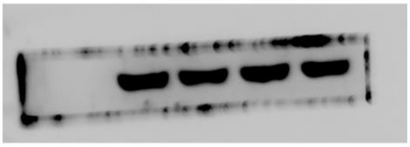 36kDa

GAPDH-marker 35kDa---- 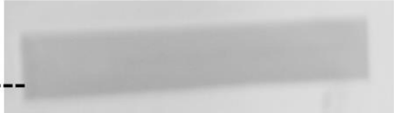

Figure 5G

P21 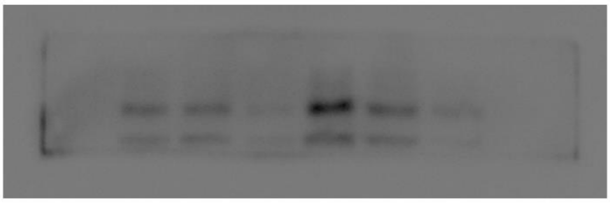 21kDa

P21-marker 15kDa-- 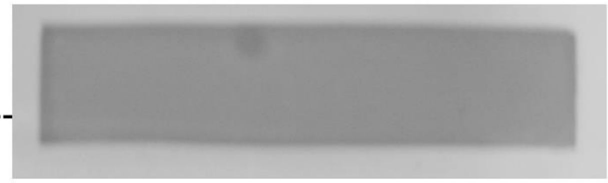

GAPDH 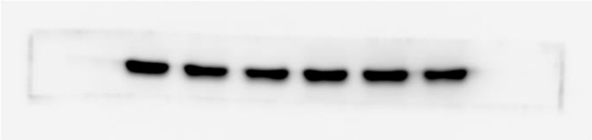 36kDa

GAPDH-marker 35kDa---- 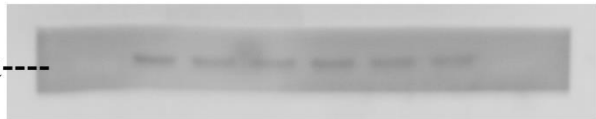

Figure 6A

P-AMPK 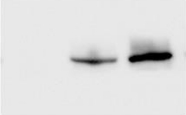 62kDa

P-AMPK-marker 70kDa----
55kDa---- 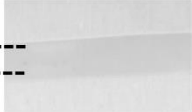

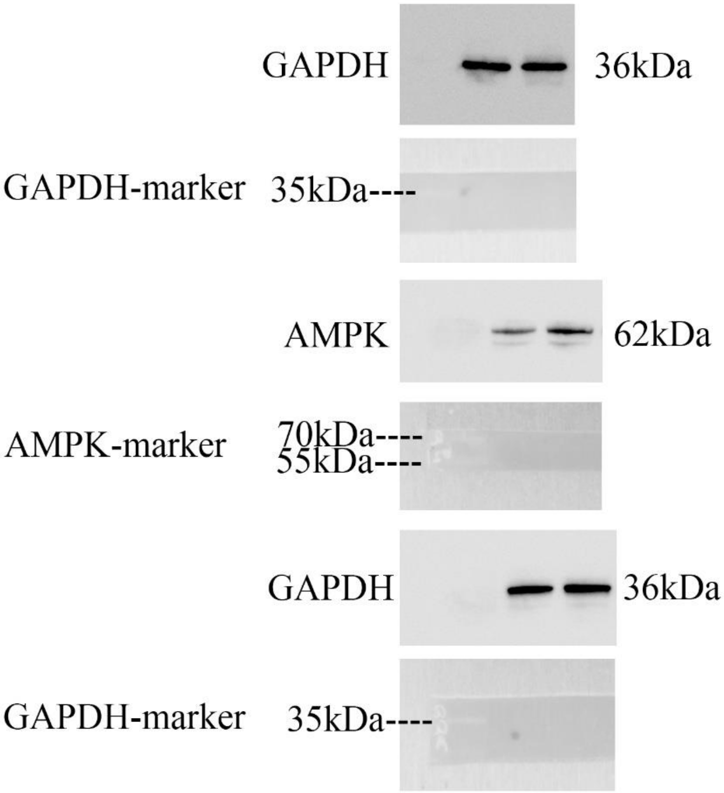
GAPDH
36kDa
GAPDH-marker
35kDa----
AMPK
62kDa
AMPK-marker
70kDa----
55kDa----
GAPDH
36kDa
GAPDH-marker
35kDa----

Figure 6B

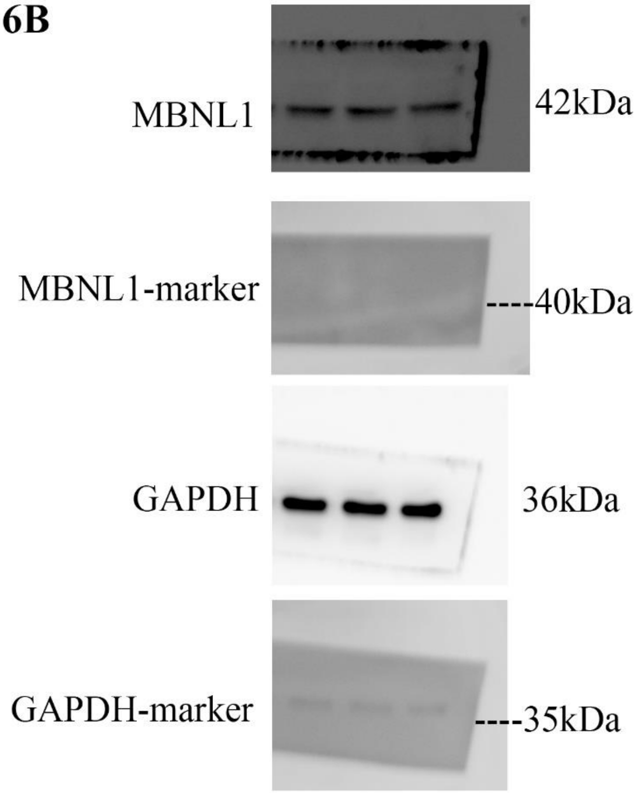
MBNL1
42kDa
MBNL1-marker
----40kDa
GAPDH
36kDa
GAPDH-marker
----35kDa
